# Shifts in bee diet breadths are associated with gene gains and losses and positive selection across olfactory receptors

**DOI:** 10.1093/g3journal/jkaf105

**Published:** 2025-07-11

**Authors:** Avehi Singh, Nathaniel S Pope, Margarita M López-Uribe

**Affiliations:** Intercollege Graduate Degree Program in Ecology, The Pennsylvania State University, University Park, Pennsylvania 16803, USA; Department of Entomology, The Pennsylvania State University, University Park, Pennsylvania 16803, USA; Institute of Ecology and Evolution, University of Oregon, Eugene, Oregon 97403, USA; Intercollege Graduate Degree Program in Ecology, The Pennsylvania State University, University Park, Pennsylvania 16803, USA; Department of Entomology, The Pennsylvania State University, University Park, Pennsylvania 16803, USA

**Keywords:** diet, olfaction, pollination, specialist, generalist, chemosensory receptors, comparative genomics

## Abstract

Bees are palynivorous insects that vary widely in the number of plant families from which they collect pollen. Their evolutionary history has been marked by multiple transitions in diet breadth between specialists that only visit specific plant genera (narrow diet breadth) and generalists that visit multiple large plant families (broad diet breadth). Understanding the evolution of sensory systems associated with changes in the detection, discrimination, and gustation of different pollen in bees can shed light on the underlying genetic mechanisms associated with transitions between narrow and broad diet breadths. We conducted a comparative study of three families of insect olfactory receptor genes (odorant receptors (ORs), gustatory receptors (GRs), and ionotropic receptors (IRs)) linked to diet breadth across 51 bee species. We calculated rates of gene gains and losses and identified genes experiencing positive selection across specialist and generalist bees. Our results show that broad generalists exhibit high rates of OR gene losses and GR gene gains. We observed accelerated rates of evolution in seven orthologous groups of genes across specialists and one group in generalists. Several orthogroups showed diversification in putative ligand-binding domains of proteins, indicating potential shifts in functional properties of the receptors. Taken together, these results indicate that dietary specialization in bees requires chemosensory system diversification of existing genes while dietary generalization is associated with the loss of ORs and gain of GRs. Our study provides important insights into the genetic architecture underlying shifts in niche occupancy across insects.

## Introduction

Bees are a globally distributed group of obligate palynivorous insects that rely on pollen for their growth and development. Their diet breadths vary widely, ranging from a single plant species to several plant families. The earliest bees ∼120 MYA were likely diet specialists, collecting pollen from a few plant species; but the radiation of Angiosperms was coupled with the diversification of bees and the emergence of diet generalization (Cardinal and Danforth 2013; Wappler *et al*. 2015). Subsequently, the evolutionary history of bees has involved multiple shifts between specialization and generalization in pollen collection (Dorchin *et al*. 2021). These shifts in bee diet breadths have in turn been linked to the diversification of multiple plant families and likely contribute to structuring ecological networks globally (Minckley *et al*. 2000). Despite the importance of bee diet breadths in maintaining plant community diversity and function globally, little is known about the underlying genetic mechanisms behind the evolution of bee dietary breadth.

Changes in diet breadths of palynivores over their evolutionary history likely involve shifts in their ability to locate and discriminate between different floral resources. Bees, like most insects, have particularly well-developed olfactory systems, which are their primary sensory modality for long-range detection of floral resources and nutrient discrimination (Bohbot *et al*. 2007; Howell and Alarcón 2007; Ramírez *et al*. 2011). A key set of proteins involved in insect olfaction are olfactory receptors—ion channel proteins located on the surfaces of sensory neurons housed within sensillar hairs located on mouthparts and olfactory organs. Chemosensation occurs when chemical cues from the environment enter the sensillar lymph via pores on the surface, bind to chaperone proteins that transport them to the neuronal surface, and interact with olfactory receptor complexes to induce neural activity (Hansson and Stensmyr 2011). Thus, these receptors are crucial components of peripheral sensory systems in bees as they form the link between the environment and the neural systems of these insects.

Insect olfactory receptors are subdivided into three gene families: ionotropic receptors (IRs), gustatory receptors (GRs), and odorant receptors (ORs) (Andersson *et al*. 2015). IRs are an ancient, conserved group of receptors across insects. Only a few IRs are involved in olfaction (Croset *et al*. 2010; Ni, 2021) and are known to respond to water-soluble volatile odorant compounds (including acids, alcohols, and amines) in *Drosophila melanogaster*. However, little is known about their function in other insect families (Dhakal *et al*. 2021). GRs are heterotetrameric protein complexes with several conserved coreceptor proteins present across Eukaryotes, although their chemosensory function might be specific to Arthropoda (Robertson 2015). These receptors are mainly involved in detecting gustatory (such as sugars and amino acids) and a few odorant (such as CO_2_) stimuli. GRs often operate over short spatial distances, as they mainly detect nonvolatile compounds and prompt appetitive and aversive responses. ORs are a large family of genes that likely originated from GRs during the emergence of Insecta (Brand et al. 2018). Like GRs, ORs form heterotetrameric complexes with a conserved coreceptor protein but differ in their structural and functional properties (Hansson and Stensmyr 2011). Insect ORs have been shown to respond to a wide variety of volatile odorant cues and are often sensitive to low cue concentrations, allowing them to operate over large spatial scales (Bohbot and Pitts 2015).

Prior studies comparing evolutionary patterns in olfactory systems across insect diet breadths suggest that complex evolutionary processes within and across gene families, such as gene losses and protein evolution, are associated with shifts between generalist and specialist diets. A well-studied example is that of the specialist *Drosophila sechellia*, a subspecies of *Drosophila simulans* which is narrowly specialized on ovipositing on Noni fruit (*Morinda citrifolia*). The evolutionary history of *D. sechellia* indicates that it experienced high rates of OR and GR losses coupled with the expansion and diversification of specific OR genes required for both short- and long-range detection of Noni fruit (Auer *et al*. 2021). Studies investigating evolutionary changes in olfactory systems associated with shifts from specialized to generalized diets in the chelicerate *Tetranychus urticae* and the fly *Bactrocera dorsalis* show that generalization was accompanied by increases in OR and GR gain rates and consequently, these species express more receptors than related specialists (Ngoc *et al*. 2016; Wang *et al*. 2022). ORs have expanded and diversified in bees compared to the Apoid wasps from which they originated (Robertson and Wanner 2006; Obiero *et al*. 2021) and functional studies have linked this diversity to multiple olfaction-dependent life history traits in bees such as associations with flowers, chemical communication in social species, and mating behavior (Zhou *et al*. 2015; Brand and Ramírez 2017; Brand *et al*. 2020). Conversely, bees have a relatively reduced GR repertoire compared to other Hymenopterans and Dipterans, which may reflect a relative paucity of gustatory cues used by these insects (Bestea *et al*. 2021; Obiero *et al*. 2021).

We conducted a broad comparative study of olfactory receptor gene evolution across bee specialists and generalists. We investigate two competing hypotheses describing how the evolution of olfactory receptor gene families is linked to dietary specialization and generalization in bees. Specifically, our first hypothesis states that specialists show diversification in OR and GR genes to specifically bind to floral compounds in host flowers alongside losses in ORs, GRs, and IRs that are not specific to these cues (as shown in Goldman-Huertas *et al*. 2015; Auer *et al*. 2020; Matsunaga *et al*. 2022). However, since specialization is the ancestral dietary state for bees, an alternative hypothesis predicts that specialists may not lose genes, rather, shifts to diet generalization may be associated with expansions and the rapid evolution of OR and GR genes (as shown in Ngoc *et al*. 2016; Wang *et al*. 2022). To test these hypotheses, we used a sample of 51 bee genomes spanning a spectrum of diet breadth from narrow specialists to broad generalists grouped into four categories of diet breadth. We generated annotations for OR, GR, and IR genes across all the genomes sampled simultaneously and contrasted rates of gains and losses across specialists and generalists. We then identified orthologous groups of genes that experienced positive selection within diet breadth categories, generated de-novo 3D protein structures for these, and mapped diversifying residues (those showing accelerated rates of nonsynonymous substitutions) onto these models. We found that bee species with the broadest diet breadths (i.e., broad polylectic species) had higher rates of OR loss and GR gains than species with less generalist diet breadths (i.e., polylectic species). We identified eight genes that showed signatures of positive selection in specialists and generalists, several of which had diversifying amino acid residues in protein domains previously implicated in ligand-binding function. We establish links between shifts in dietary breadth and sensory system evolution in an important insect system, providing unique perspectives on the mechanisms that drive dietary niche occupancy in insects.

## Materials and methods

### Sample collection for de-novo genomic and transcriptomic datasets

**Fig. 1.**
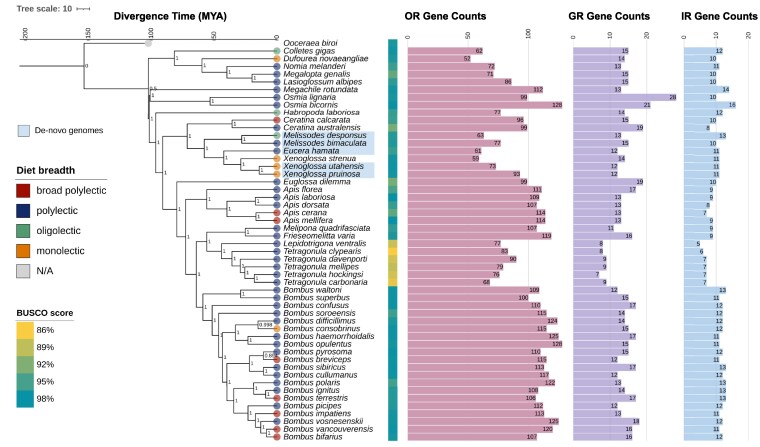
Species phylogeny with gene counts. Maximum likelihood time-calibrated ASTRAL-III coalescent phylogeny of 51 bee species generated using 5,922 single-copy orthologous genes identified using BUSCO. Genomes that were sequenced for this study are indicated using blue highlights. Numbers at each node indicate support values as inferred by CASTLES using 1,000 bootstrap intervals. We used previously validated fossils (Supplementary Table 9) to calibrate the phylogeny and dated the root of the phylogeny at ∼110 MYA, which is within the range of the inferred origin of bees at 120–150 MYA (Almeida *et al*. 2023). Genome completeness, as assessed by BUSCO scores, for each assembly are indicated by the colored boxes next to tip labels. We categorized species within our dataset into four diet breadth categories, indicated as dots at the tips: broad polylectic (*N* = 8), polylectic (*N* = 34), oligolectic (*N* = 3), and monolectic (*N* = 5) species using publicly available datasets on floral visitation and palynivory (Supplementary Table 7). Bar plots show the number of OR, GR, and IR genes annotated for each species with the values shown at the tips of the bars.

We collected 20 males for five specialist and generalist species (*Xenoglossa utahensis*, *Xenoglossa strenua, Eucera hamata, Melissodes bimaculata*, and *Melissodes desponsus*) within the tribe Eucerini (Hymenoptera: Apidae) for whole genome sequencing (WGS) (indicated as highlighted genomes in Fig. 1 and see Supplementary Table 1 for sample collection information). We chose to sample male bees as they are haploid and thus allow us to avoid heterozygosity across alleles. Bees were hand-collected on flowers at the Arboretum at Penn State University in University Park, PA, USA (40°48′48.0788″ N, 77°52′37.984″ W) and the Penn State Fruit Research and Extension Center in Biglerville, PA, USA (39°56′6.493″ N, 77°15′19.288″ W) between May and July 2019–2021. Species ID was performed with assistance from Shelby Kilpatrick, Dr. Stephania Sandoval, and Dr. David Biddinger and voucher specimens were deposited in the López-Uribe laboratory collection. All other samples were flash-frozen in liquid nitrogen or transferred into 1.5 ml RNA*later*™ Stabilization Solution (ThermoFisher; Waltham, MA, USA) and stored at −80°C.

### WGS datasets

Total gDNA was extracted for three males per species using a modified CTAB extraction protocol (see Supplementary Table 2 for quality of gDNA extractions). The eyes of each bee were removed using sterilized stenotomy scissors, the abdomens were retained as vouchers, and the head and thorax were homogenized in Gilbert buffer with 10 µ*l* 100 mg/ml Proteinase K. After overnight incubation at 55°C in a shaking water bath, we performed phase separation by adding 100 µ*l* phenol:chloroform:isoamyl (25:24:1), centrifuging at 16,000 g for 5 min, and isolating the aqueous phase. DNA was precipitated by adding 20µ*g* glycogen, 0.6 vol isopropanol, and 0.1 vol 3 M sodium acetate then centrifuging at 16,000 g for 25 min at 4°C and discarding the supernatant. The pellet was washed twice with 85% ethanol at −20°C, dried with a vacuum centrifuge, and suspended in 100 µ*l* molecular grade water. DNA concentration was quantified using Qubit dsDNA high sensitivity kit (Invitrogen; Carlsbad, CA, USA) and Spectramax id3 plate reader (Molecular Devices; San Jose, CA, USA). Based on DNA concentration and purity, we selected one sample per species for sequencing at 60× coverage using the Illumina Miseq v2 platform to generate 150 bp paired-end reads (Novogene Corporation Incorporated; Sacramento, CA, USA). We removed adaptor sequences and duplicates and then trimmed the raw reads with BBduk v.35.85 (Bushnell *et al*. 2017) using the following parameters: *hdist* = 1, *ktrim* = r, *k* = 23, *mink* = 11, *qtrim* = rl, *trimq* = 10, *tbo* = t, *tbe* = t, and *minlen* = 70.

### Antennal RNA-Seq datasets

We generated pooled antennal transcriptomes for four out of the five species sampled (see Supplementary Table 3 for quality of RNA extractions). We dissected antennae for ten males per species using sterilized forceps and thawed the tissue in 1 *ml* TRIzol reagent (Invitrogen; Carlsbad, CA, USA). Samples were homogenized using 5–6 beads from a BeadBashing lysis kit (Zymo; Irvine, CA, USA) in two cycles of 30 s at 7 m/s in a bead-beater homogenizer (Molecular Devices; San Jose, CA, USA). The lysate was centrifuged at 24,000 g at 4°C for 10 min, the supernatant was transferred into a new tube, and incubated for 5 min at room temperature. Phase separation was performed by adding 200 µ*l* chloroform, vortexing, incubating for 2–3 min, centrifuging at 12,000 g at 4°C for 15 min and transferring the aqueous phase. Total RNA was precipitated by adding 250 µ*l* isopropanol, 250 µ*l* of 1.2 M NaCl, and 0.8 M Na_3_C_6_H_5_O_7_ then incubating for 10 min, centrifuging at 12,000 g at 4°C for 10 min, and discarding the supernatant. The RNA pellet was washed with 1 *ml* of 75% ethanol, air dried, and suspended in 130 µ*l* molecular grade water. DNA contaminants were removed by adding 50 µ*l* 8 M LiCl, incubating overnight at 4°C, centrifuging at 10,000 rpm at 4°C for 15 min, and discarding the supernatant. The RNA pellet was incubated with 100 µ*l* molecular grade water, 150 µ*l* ethanol, and 10 µ*l* 3 M sodium acetate for 2 h at −20°C. Samples were centrifuged at 14,000 rpm at 4°C for 15 min, washed with 1 *ml* 75% ethanol solution twice. The pellet was air dried and solubilized in 20 µ*l* RNAse-free water. RNA quality was assessed with Qubit RNA high-sensitivity kit (Invitrogen; Carlsbad, CA, USA) and Spectramax id3 plate reader (Molecular Devices; San Jose, CA, USA). Total RNA was sequenced to 30× depth using an Illumina Novaseq 6000 platform to generate 150 bp paired-end reads (Novogene Corporation Incorporated; Sacramento, CA, USA). The produced reads were quality-filtered and sequencing and indexing adapters were removed using Trimmomatic v.0.32 (Bolger *et al*. 2014).

### Publicly available datasets

We queried the National Center for Biotechnology Information (NCBI) Sequence Read Archive (SRA) database for available WGS datasets for bee species (*N* = 49) alongside data for the clonal raider ant *Ooceraea biroi* (accessed February-March 2021) using the SRA Toolkit v.2.10.0 command line functions (Leinonen et al., 2011) (see Supplementary Table 4 for SRA accession information and citations for previously generated datasets used).

### Genome assembly

Sequencing data generated for this project is available under NCBI Project number PRJNA1095984. All WGS datasets were assembled using the same pipeline to standardize genome completeness and control for differences in sequencing effort across species. We used the SPAdes v.3.15.0 assembler (Prjibelski *et al*. 2020) at default settings to assemble de-novo genomes. We performed contig correction by using the RagTag v.2.1.0 correction module (Alonge *et al*. 2022) using the chromosome-scale *Xenoglossa pruinosa* genome (Pope *et al*. 2023) as a reference. We then used the RagTag scaffold module to orient contigs into scaffolds and find sequencing gaps. We assessed levels of contamination by filtering out contigs with coverage ≥4 reads and length ≥1000 bp. We removed contaminant sequences using minhash as implemented in the “*sendsketch.sh*” script in the BBtools suite (Bushnell *et al*. 2017). Next, we used Minimap v.2.20 (Li, 2016) to map raw reads back onto the assembled genomes, SAMtools v.1.13 (Li *et al*. 2009) to sort and index the alignments, and finally either NextPolish v.1.4.0 (Hu *et al*. 2020) or Pilon v.1.24 (Walker *et al*. 2014) (in the case of *E. hamata*) to polish the genome with three rounds of polishing. We closed gaps in the genome using Abyss-Sealer v.0.6.0 (Paulino *et al*. 2015) setting k-mer values to 48, 64, 80, 96, 112, and 128 and a 150 bp flank size for pseudoreads. Finally, we assessed genome quality using QUAST v.5.1.0 (Gurevich *et al*. 2013) and BUSCO v.5.5.0 (Manni *et al*. 2021) run using the hymenoptera_odb10 dataset trained on the “*honeybee1*” species dataset (see Supplementary Table 5 for genome quality metrics).

### Phylogenetic inference

We used the single copy orthologs (*N* = 5,922) identified in each genome as part of the BUSCO pipeline to construct a species tree. Nucleotide sequences were translated to amino acid sequences that were used to generate alignments per gene using the E-INS-i method in MAFFT v.7.182 (Katoh and Standley 2013), trimmed using heuristic algorithms implemented in trimAL v.1.2 (Capella-Gutiérrez *et al*. 2009) using the “*automated1*” setting, and used as input for gene tree estimation in IQTree v.2.3.2 using ModelFinder v.1.7.5 with 1,000 ultrafast bootstrap replicates (Kalyaanamoorthy *et al*. 2017 ; Minh *et al*. 2020) to estimate best-fit partition schemes and substitution models per gene family. The resulting gene trees were used as input to ASTRAL-III v5.7.1 (Mirarab *et al*. 2014) to produce a coalescent-based species tree rooted on *O. biroi*. Finally, we used the Coalescent-Aware Species Tree Length Estimation in Substitution-unit method implemented in CASTLES v.1.3.0 (Tabatabaee *et al*. 2023) to estimate branch lengths within the ASTRAL-III topology using the BUSCO gene trees as input.

To estimate divergence times across the species phylogeny, we first used a penalized likelihood method implemented in the function “*chronos*” implemented in the package ape v.5.7.1 (Paradis and Schliep 2019) using previously published fossil records (see Supplementary Table 9 for informations on fossils used as calibration points). We used the “*makeChronosCalib()*” function to assign minimum and maximum ages for each calibration point based on prior paleontological evidence (Supplementary Table 9). We first estimated the best-fit λ, the penalized likelihood smoothing parameter, for the tree using the “*chronopl*” function in ape. We then tested multiple clock models, including strict, relaxed, and autocorrelated rate models, by varying the lambda parameter. Model fit was assessed using log-likelihood values and Akaike Information Criterion (AIC). The topology of the best-fit phylogeny was visually compared with previously published bee phylogenies to ensure congruence with known phylogenetic relationships within bee families (Branstetter *et al*. 2017; Peters *et al*. 2017; Sun *et al*. 2021). We used iTOL v.6 (Letunic and Bork 2021) to generate Fig. 1 and Supplementary Figs. 1–3.

### Genome alignment and annotation

We used Repeatmodeler v.2 to generate ab initio repeat libraries for each genome and Repeatmasker v. 2.11.0 (Chen 2004) and redmask v.0.0.2 (Girgis 2015) (in the case of the de-novo genomes) to softmask all the genomes. We then used ProgressiveCactus v.1.2.3 (Armstrong *et al*. 2020) to align all genomes in this study using the species phylogeny as a guide. Due to issues with the availability of computational resources, we broke the species phylogeny up into 15 clades subtending tips and ancestral nodes across the phylogeny. Each of these clades was aligned separately while progressively moving up the tree, using default settings with the generated species phylogeny to guide the alignment. We assessed the completeness of the produced HAL alignment using the `*halstats.sh*` script within ProgressiveCactus.

We used a comparative approach to annotate genes for all three receptor families in the produced genome alignment, based on pipelines developed by (Fiddes *et al*. 2018). For each gene family, we built a custom library of protein and nucleotide models by querying the NCBI refseq database for genes previously annotated across insect species and aligning protein sequences using Exonerate v.2.2 (Slater and Birney 2005). Antennal RNA-seq datasets were mapped back to the *X. pruinosa* genome using STAR v.2.4.0 (Dobin *et al*. 2013). We used the “exonerate2hints.pl” script and bam2hints in AUGUSTUS v.3.4.0 (Stanke *et al*. 2006) to generate hints files for each gene family separately. We exported the HAL alignment into a SQLite database and used MetaEuk v.5 (Levy Karin *et al*. 2020) to predict intervals within the alignment putatively containing olfactory receptor genes. We merged overlaps within intervals using BEDtools 2.28.0 (Quinlan and Hall 2010), constructed GFF files for each prediction with a 5 kB buffer on either end and mapped these back onto the HAL alignment to generate aligned intervals. Finally, we ran AUGUSTUS on “*cgp*” mode (Nachtweide and Stanke 2019) on each aligned interval using the generated hints files to generate putative gene annotations. The “*joingenes*” script in AUGUSTUS was used to concatenate genes per species into GFF files containing amino acid predictions. This initial set of gene models was filtered by removing sequences that were >200 amino acids long and used TMHMM v.2 (Krogh *et al*. 2001) to further remove sequences that had fewer than three predicted transmembrane regions.

### Identification of orthologous genes across species

We aligned the filtered gene sequences for each gene family using the E-ins-I method in MAFFT, manually removed gappy sequences from the alignment, and removed regions within the alignment that contained >70% gaps using Trimal. Maximum likelihood gene trees were inferred using IQTree with 1,000 ultrafast bootstrap replicates, using ModelFinder to find the best fit substitution model per gene family. Each tree was manually parsed into orthogroup clusters on the TreeViewer v.2.2.0 server (Bianchini and Sánchez-Baracaldo 2024), identifying the MRCA per cluster, and using the “*keep.tip*” function in the package phytools v.2.1.1 (Revell 2024) in R (v.4.4.2; R Core Team 2024) to generate aligned sequences per cluster (see Supplementary Figs. 1–3 for phylogenies of each gene family with delineated orthogroups). We were not able to manually identify orthogroups within the OR gene tree as it was too large and complex (See Supplementary Fig. 1 for the phylogeny of ORs). To simplify this problem, we used the “*as.hclust*” function in base R v.4.4.2 to break the tree into smaller subtrees and manually parsed each subtree. All orthogroup subtrees were mapped back onto the original gene trees to manually remove overlaps and outliers.

We used protein alignments for each orthogroup as input for HMMbuild (hmmer.org) to build a hidden Markov model (HMM) profile per orthogroup. These models were used to filter the corresponding nucleotide alignments using the HMMalign program. We removed insertion sequences from the filtered alignments using bioawk and trimmed them with trimAL run using the “*gappyout*” setting. We then used IQTree run using ModelFinder to generate the best-fit maximum likelihood phylogeny per orthogroup, using an alignment of custom libraries of *Drosophila melanogaster* sequences as an outgroup. Finally, each tree was manually parsed to remove misplaced tips and the filtered protein trees were used as input to downstream analyses.

### Diet breadth classification

We used publicly available records and published plant–pollinator networks (see Supplementary Table 7 for sources used to assign diet breadth per species) to assign species into four categories of diet breadth: broad polylectic (species that collect pollen from >25% of available plant families), polylectic (species that collect pollen from ≥4 plant families), oligolectic (species that collect pollen from 1-3 plant families), and monolectic (species that collect pollen from a single plant family or genus). We used definitions of oligolecty, polylecty, and broad polylecty based on those in Cane and Sipes (2006) and the revised definition of monolecty by Cane (2021). We excluded three genomes from downstream analyses as they represented two kleptoparasitic species that do not forage for pollen and one species for which we could not obtain reliable dietary information. We thus split our dataset into eight broad polylectic species, 35 polylectic species, three oligolectic species, and five monolectic species (Methods and Supplementary Table 7).

### Ancestral state reconstruction of diet breadth

We inferred ancestral diet breadths across the species tree using the “*fitMK*” function in the package Phytools v.2.4.4 (Revell 2024). We tested three models of trait evolution (ER: equal rates, SYM: forwards and reverse transitions share parameters, and ARD: each rate is a unique parameter), estimated the root state probabilities using the fitzjohn method, which accounts for uncertainty in the root state, and identified the best-fit model based on AIC values. We then used the function “*ancr*” in Phytools to estimate the probability of each state at each internal node within the tree, marginalizing over all possible states at other nodes (see Supplementary Table 8 for estimated diet breadth probabilities at nodes).

### Gene gain and loss rate analysis

CAFE v.5 assumes a birth-death model of gene evolution and estimates a parameter λ, the mean probability of a gene being gained or lost within a unit of time across the time-calibrated phylogeny (Mendes *et al*. 2020). We inferred maximum likelihood λ values for each gene family, using a matrix of gene counts per species as inputs. We first fit an error model to control for variations in genome assembly quality and annotation errors. We then used CAFE to estimate the best-fit gamma rate value for each gene family for 2–5 gamma categories using generated log-likelihood values and incorporating the error model. We performed five runs of each model using the best-fit gamma value to ensure convergence. We extracted maximum likelihood estimates of ancestral gene count and the number of gene gains and losses at each node of the phylogeny from the best-fit CAFE model for each gene family. We estimated mean gene gain and loss rates per species using differential equations described in Niimura et al. (2014) (see Supplementary Table 11 for estimated gene gain and loss counts per branch and estimated rates of gene gain and loss for each gene family).

### Molecular evolution analysis

Branch-site models of codon evolution use a likelihood-based framework to assess whether the ratio of nonsynonymous:synonymous amino acid substitutions d_N_/d_S_ (hereafter represented as ω) significantly differs across test and background branches within the gene trees of each orthogroup. For each orthogroup, we labeled filtered gene trees to assign (1) broad polylectic species, (2) oligolectic species, (3) monolectic species, and (4) monolectic and oligolectic species as foreground branches.

Aligned nucleotide sequences for each orthogroup were translated into protein sequences using Pal2nal v.14 (Suyama *et al*. 2006) assuming the universal genetic code (NCBI: transl_table = 1). We trimmed each codon alignment to remove sites with 80% gaps as well as residues present in less than 75% of the sequences using trimAL. This filtering step eliminated four OR orthogroups (orthogroups 9, 12, 15, and 30) and we omitted them from further analysis.

For each set of labeled gene trees and corresponding codon alignments, we ran branch-site models in codeml implemented in PAML v.4.10.7 (Yang 2007; Álvarez-Carretero *et al*. 2023) to test for positive selection in foreground branches (see Supplementary Table 15 for results of branch-site tests). Briefly, codeml fits two models: the null model assumes in the foreground branches was fixed at 0 and the alternative model allows ω > 1 in the foreground branches using a 50:50 mixture of 0 and *χ*^2^ as the null distribution. We used the following settings in the codeml control file: the null model (model = 2, NSsites = 2, fix_omega = 1, omega = 1, CodonFreq = 2) and the alternative model(model = 2, NSsites = 2, fix_omega = 0, CodonFreq = 2). We then used a likelihood ratio test to identify the best-fit model.

Branch-site models in PAML do not distinguish between phylogeny-wide positive selection and selection restricted to specific branches. Thus, we also used the input files for codeml to run branch-site unrestricted statistical tests for episodic diversification—phylogeny (BUSTED-Ph) (Murrell *et al*. 2015) implemented in HYPHY v.2.5.21 (Kosakovsky Pond *et al*. 2020) (see Supplementary Table 16 for results of BUSTED-Ph analyses). This approach also tests for sites in background branches where ω > 1 and incorporates this evidence into the likelihood-based framework to ascertain whether diversification is only occurring in foreground branches. To assign potential functional and structural identities to orthogroups identified by both methods, we conducted extensive tBLASTn searches to identify orthologous proteins within the NCBI refseq database (See Table 1 and Supplementary Table 17 for identified orthologous genes).

**Table 1.** Joint identification of olfactory receptor gene orthogroups experiencing positive selection in specialists and generalists using codeml branch-site models and BUSTED-Ph tests implemented in HYPHY. Paritions listed are species within the references diet breadth categories. In all cases an additional partition exits that contains the remaining taxa (referred to as the background in both sets of models). The table provides estimated ω values for discrete site classes alongside the proportaion of sites for each class, estimated log-likelihood scores, and FDR-corrected *P* values for both constrained and unconstrained models.

Partition	Gene	Orthogroup	PAML branch-site										HYPHY BUSTED-Ph						
			Model	np	lnL	K	ω0/p0	ω1/p1	ω2a/p1a	ω2b/p2b	2Δl	*P*	AIC-c	lnL	Model	ω0/p0	ω1/p1	ω2/p2	*P*
Monolectic + Oligolectic	IR	9	M0	101	−19531.204	4	0.160/0.646	1/0.318	2.1/0.0242	2.1/0.119	6.552	0.0105	52842.161	−26294.577	null	0/0.0128	0.167/0.917	3.489/0.0698	3.22E-11
			M2	102	−19527.928		0.156/0.601	1/0.291	1/0.0726	1/0.0351					alt	0.000716/0.606	1/0.392	2799.914/0.00182	
Monolectic + Oligolectic	GR	7	M0	143	−10896.34	4	0.195/0.534	1/0.412	2.1/0.030	2.1/0.0231	4.0951	0.043	42382.621	−21011.688	null	0/0.362	0.699/0.600	10.762/0.0388	0.0331
			M2	144	−10894.292		0.120/0.555	1/0.43	15.044/0.00826	15.044/0.00641					alt	0.0518/0.375	0.127/0.279	1.901/0.346	
Monolectic	GR	1	M0	79	−241.029	4	0/0.167	1/0.83	2.1/0	2.1/0	18.145	2.05 E-05	9891.6678	−4848.158	null	0.0554/0.871	1/0.127	135.742/0.00148	0.0297
			M2	80	−231.956		0.0733/0.84	1/0.16	1/0	1/0					alt	0/0.631	0.00397/0	5.078/0.369	
Monolectic	GR	8	M0	93	−1151.173	4	0/0	1/0.783	2.1/0	2.1/0.217	5.717	0.0168	56021.105	−27,895	null	0/0.378	1/0.608	198.333/0.0139	3.29E-05
			M2	94	−1148.314		0.575/0.329	1/0.412	12.97/0.115	12.97/0.144					alt	0.0218/0.653	1/0.00887	2.951/0.338	
Broad Polylectic	OR	37	M0	233	−252.299	4	0/0	1/0	2.1/0	2.1/0	6.0394	0.014	76230.613	−37859.914	null	0/0.00441	0.167/0.916	4.446/0.0798	1.98E-13
			M2	234	−249.279		0/0	1/1	1/0	1/0					alt	0.0000077/0.719	0.366/0.251	19.243/0.0309	
Monolectic + Oligolectic	OR	3	M0	123	−4087.464	4	0.0813/0.713	1/0.221	2.1/0.0507	2.1/0.0157	9.619	0.00193	35963.996	−17835.118	null	0/0.711	0.705/0.288	953.583/0.00156	3.27E-07
			M2	124	−4082.655		0.0748/0.634	1/0.191	1/0.135	1/0.0406					alt	0/0.599	0.743/0.374	17.750/0.0267	
Monolectic + Oligolectic	OR	4	M0	125	−14425.237	4	0.139/0.672	1/0.213	2.1/0.0873	2.1/0.0277	23.93	9.99 E-07	37406.396	−18557.346	null	0.0492/0.790	1/0.209	286.594/0.00111	3.30E-06
			M2	126	−14413.272		0.130/0.577	1/0.178	1/0.187	1/0.0579					alt	0/0.0482	0.230/0.875	5.431/0.0767	
Monolectic + Oligolectic	OR	24	M0	101	−4397.905	4	0.0995/0.624	1/0.284	2.1/0.0637	2.1/0.0290	6.418	0.0113	19244.579	−9506.172	null	0/0.509	0/0.192	0.922/0.299	0.0174
			M2	102	−4394.696		0.0921/0.552	1/0.256	1/0.131	1/0.0607					alt	0.0752/0.719	0.109/0.126	2.668/0.155	

### Protein structure analysis

We generated consensus protein sequences for each of the eight orthogroups identified using the EMBOSS toolkit (Rice *et al*. 2000) and used these as input to the MMseqs2 implementation of Alphafold2 v.2.3.2 run on the Google Colab server (Bryant *et al*. 2022; Mirdita *et al*. 2022) to generate structural models. We generated five structural models for each orthogroup and used the structure with the highest confidence score as assessed by predicted template modeling (pTM) score for codon mapping (see Figs. 3 and 4 and Supplementary Figs. 4 and 5 for structures generated for each orthogroup). We assessed the quality of the structural models we obtained through pTM and predicted local distance difference test (pLDDT) scores obtained through AlphaFold2. We also implemented the tools ERRAT, PROCHECK, and VERIFY 3D on the UCLA-DOE LAB—SAVES server v6.1 (https://saves.mbi.ucla.edu/) to generate additional metrics of structural quality (see Supplementary Table 18 for quality metrics for obtained structures). We accessed pdb structures of previously generated monomeric models of AbOrco (pdb ID: 6C70), BmGR9 (pdb ID: 8UVT), and a tetrameric model of AMPA subtype ionotropic glutamate receptor (pdb ID: 3KG2) from the protein data bank (PDB) database to use as a comparison set for our de-novo structural models (Sobolevsky *et al*. 2009; Butterwick *et al*. 2018; Gomes *et al*. 2024). We then used TM-align and PyMOL v.2.6 (DeLano Scientific; San Francisco, CA, USA) to compare structures of de-novo and reference structural models (Zhang and Skolnick 2005) and calculated root mean squared deviation (RMSD) values between pairs of atoms.

To identify which protein domains were enriched for diversifying amino acid residues within genes experiencing positive selection in specialists and generalists, we used fixed effects site-level models implemented in FEL-CONTRAST models in HYPHY v.2.5.21 (Kosakovsky Pond et al. 2021) setting the adjusted *P* value threshold as 0.05 (see Supplementary Table 19 for results of FEL-CONTRAST models). While both branch-site models in codeml and BUSTED-Ph identify codons that are experiencing diversification within foreground branches, both methods use empirical Bayesian approaches that may not be accurate. FEL-CONTRAST is a frequentist approach that is therefore better suited to accurately identify individual sites where ω > 1 in pre-specified foreground branches. We used the input codon alignments and labeled gene trees used for BUSTED-Ph analyses for this analysis. We mapped diversifying codons onto the 3D structures using PyMOL and used the plugin “*pymol-color-alphafold*” to color the protein structures by pDDLT values.

### Statistical testing

All statistical analyses were conducted in R version 4.4.2 (R Core Team (2024)). To control for phylogenetic relatedness, we assessed the significance of phylogenetic signal in diet breadth by calculating Blomberg’s K statistic across diet breadth categories using the “*phylosig*” function in the package phytools v.2.4.4 (Revell 2024) and conducting a permutation test with 1,000 iterations to assess the statistical significance of the observed K value. To evaluate the strength of phylogenetic signals in continuous variables considered within this study (gene repertoires, gain rates and loss rates for each gene family), we used the “*phylosig*” function to conduct likelihood ratio tests to test the probability of Pagel’s λ > 1 (see Supplementary Table 12 for estimated Pagel’s lambda values).

We fit phylogenetic generalized least squares (PGLS) models using the “*gls*” function in the package nlme v.3.1–166 (Pinheiro *et al*. 2025) with phylogenetic covariance structures specified within the package ape v.5.7.1 (Paradis and Schliep 2019). For each test, we identified the best model by fitting a series of nested PGLS models to identify which variables should be included within the model formula using the AIC. After specifying model structures, we identified the best-fit phylogenetic covariance structure by comparing PGLS regressions assuming (1) Brownian motion (2) Pagel’s Lambda, and (3) Blomberg’s K models of trait evolution and identifying the best-fit correlation structure using AIC values. Final model parameters were estimated using maximum likelihood by setting the flag (method = “ML”). Model significance was assessed using Type II ANOVA implemented in the package car v.3.1–3 (Fox and Weisberg 2019) and post-hoc comparisons were conducted using Tukey’s HSD tests in the function “*glht*” in multcomp v.1.4–28 package (Hothorn *et al*. 2008). Model residuals were visually checked for normality and homoscedasticity to validate statistical assumptions (see Supplementary Table 13 for estimated PGLS model coefficients).

As our dataset contains an overrepresentation of polylectic, corbiculate species, we re-ran the phylogenetic signal calculations and all PGLS models using a pruned dataset of 35 species with single species representatives of genera that are dominated by polylectic species (such as *Tetragonula*, *Apis*, and *Bombus*) (see Supplementary Table 14 for species used within the dataset and re-estimated model coefficients).

## Results

### A comprehensive survey of 51 bee genomes

We generated a comprehensive dataset of 51 bee genomes by collating de-novo WGS datasets generated for five paired pollen specialist and generalist species (See Supplementary Tables 1 and 2) with 46 short-read WGS datasets for bee species previously deposited on the NCBI database (Fig. 1 and Supplementary Table 4). We used the ant species *O. biroi* to use as an outgroup, as ants are some of the closest extant relatives of Apoidea that have genomic data available. Our samples spanned a large part of the evolutionary history of bees with representatives from four out of seven extant bee families (Apidae, Colletidae, Megachilidae, and Halictidae).

We used 5,922 single-copy Hymenopteran genes across species to generate a coalescent species tree spanning ∼120 MYA of evolutionary time (Fig. 1). The species tree topology recapitulates previously established relationships between bee families, with Apidae and Megachlidae as sister groups and Colletidae as sister to Halictidae (Branstetter *et al*. 2017; Peters *et al*. 2017). We calibrated this phylogeny using validated fossil records previously used to date phylogenies constructed for bees and other Hymenopterans (Supplementary Table 9). The most recent common ancestor of Formicidae and Apoidea originated ∼162 MYA, and we used this fossil to date the root of our phylogeny (Peters *et al*. 2017).

We built high-quality assemblies for all the genomes, as assessed by the Benchmarking Universal Single-Copy Orthologs (BUSCO) v.5.50 (Manni *et al*. 2021) pipeline (Fig. 1 and Supplementary Table 5). BUSCO scores for all genomes ranged between 85.9% (*Tetragonula hockingsi*) to 98.8% (*Apis mellifera*), indicating overall high levels of completeness. Six stingless bee genomes (five *Tetragonula* species and *Lepidotrigona ventralis*) showed the lowest BUSCO scores and excluding these species reduced the mean BUSCO scores to between 93.1% and 98.5%. The quality of our assemblies was comparable to previous short-read genome assemblies (Supplementary Table 6). BUSCO scores did not vary with phylogeny (Pagel’s lambda = 0.731, *P =* 0.181).

### Pollen diet breadth is evolutionarily conserved across bees

Our sample spanned a range of bee diet breadth from broad generalists (such as *A. mellifera*, which collects pollen from >100 plant families) to narrow specialists (such as *Dufourea novaeangliae*, which restricts pollen collection to a single plant species: *Pontederia cordata*) (Fig. 1). The predominance of generalist species within our dataset reflects a pre-existing bias within the existing literature, as the best-studied bee species (such as honey bees and bumble bees) are all generalists and does not reflect the true distribution of specialization within bees (Waser and Ollerton 2006). We note that the three families we are missing in this dataset (Andrenidae, Stenotritidae, and Melittidae) contain mainly specialists and including data from these groups in future studies will give clearer insights into the evolutionary processes driving pollen specialization in bees.

Ancestral trait reconstruction of diet breadth indicated that the best-fit model for the evolution of diet breadth across our phylogeny assumed equal rates of transitions between diet breadth rates, with a low overall transition rate (estimated rate = 0.00224) indicating overall conservation of diet breadth across the four bee families included within our sample (Supplementary Table 8). Further, permutation tests of Blomberg’s *K* statistic found that diet breadth categories were not strongly influenced by phylogenetic relationships across species (Blomberg’s *K* = 0.361, *P* = 0.361, permutations = 1000). PGLS models found no relationship between BUSCO scores and diet breadth, assuming a Brownian motion model (χ^2^ = 1.666, df= 3,50, *P* = 0.6445).

### Olfactory gene repertoires show phylogenetic signal and may vary across diet breadth categories

We used a comparative gene annotation approach that capitalized upon synteny information provided by multiple genome alignments to classify 4,950 ORs into 46 orthogroups, 749 GRs into 15 orthogroups, and 484 olfaction-associated IRs into nine orthogroups across 51 bee species (Supplementary Figs. 1–3). The number of genes annotated per species showed significant phylogenetic signal for all three gene families (ORs: Pagel’s lambda = 0.922, LogL = −214.488, *P* < 0.001, GRs: Pagel's lambda = 0.735, *P* < 0.001; IRs: Pagel's lambda = 0.966, *P* < 0.001) (Fig. 1). Annotated gene repertoires differed widely across bee species for ORs: the *Bombus haemorrhoidalis* genome had 125 ORs while the *D. novaeangliae* genome had 52. This variability in OR repertoire has been previously reported in bees (Forêt and Maleszka 2006; Brand and Ramírez 2017) and may be linked to diverse life history traits, such as sociality. Most of the GRs and IRs were single-copy orthologs across species, recapitulating previous findings on the relative conservation of these gene families in bees (Robertson and Wanner 2006) (See Supplementary Table 10 for a comparison of the numbers of genes annotated for this study with previous studies).

As the quality of in-silico gene annotations is highly dependent on genome completeness, we used PGLS regressions assuming a Brownian Motion covariance structure to test for relationships between gene repertoire and BUSCO scores. We found that OR and IR gene repertoires varied significantly with BUSCO scores such that we recovered more gene annotations from genomes with higher BUSCO scores (ORs: χ^2^ = 3.930, df = 1–50, *P* = 0.0475; IRs: χ^2^ = 21.138, df = 1–46, *P* < 0.001). We found no significant relationship between BUSCO score and GR annotations (χ^2^ = 2.931, df = 1–50, *P* = 0.0869) (Supplementary Table 13).

**Fig. 2.**
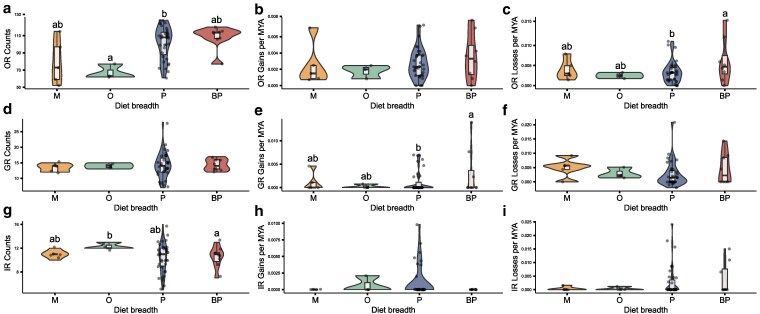
Gene counts, gain, and loss rates across diet breadth categories. Violin plots with inset bar plots showing gene counts, calculated gene gain and loss rates for three gene families across diet breadth categories. For each gene family, we used PGLS models to assess whether counts, gain,, and loss rates differed across diet breadth categories. Letters in the plots indicate pairwise significant differences across categories, as assessed by Tukey post-hoc tests. Panel (a) shows that OR counts significantly differ between oligolectic and polylectic species and panel (c) shows that OR loss rates are significantly higher in broad polylectic species compared to polylectic species. Panel (e) shows that GR gain rates are significantly higher in broad polylectic species compared to polylectic species. Panel (g) shows that oligolectic species had significantly fewer IRs than broad polylectic species. Boxes are colored by diet breadth categories as in Fig. 1 and represented by letters on the x-axis (M = monolectic, O = oligolectic, P = polylectic, and BP = broad polylectic).

We fit another set of PGLS models to understand whether gene repertoires varied across diet breadth categories with BUSCO score as an added explanatory variable for OR and IR models. Phylogenetic covariance structures within gene repertoires for all three families were best-approximated by a Brownian motion model (Supplementary Table 13). Our models showed that both OR and IR gene repertoires significantly varied across diet breadth categories (OR: χ^2^ = 9.603, df = 3–47, *P* = 0.0223; IR: χ^2^ = 9.737, df = 3–47, *P* = 0.0209). Post-hoc tests showed that, correcting for the effects of BUSCO scores, oligolectic species had significantly fewer OR genes than polylectic species (β = −22.960, SE = 9.052, *t* = −2.536, *P* = 0.0481) and significantly more IR genes than broad polylectic species (β = 2.287, SE = 0.861, *t* = 2.655, *P* = 0.0353) (Fig. 2a and c). GR repertories did not significantly vary with diet breadth categories (χ^2^ = 0.868, df = 3–47, *P* = 0.833) (Fig. 2b).

**Fig. 3.**
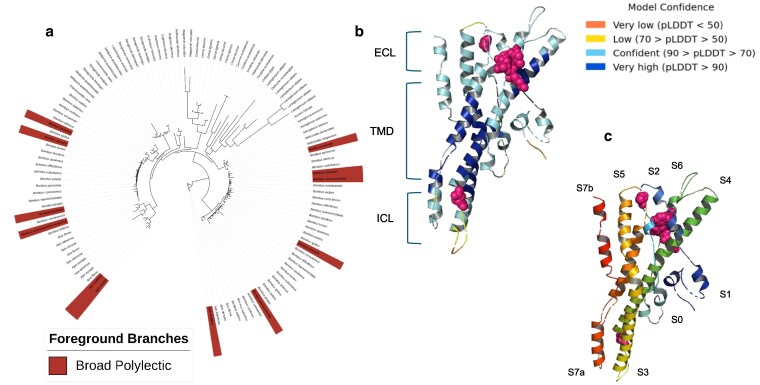
Gene tree and protein structural models for OR orthogroup 37. a) Maximum likelihood phylogeny of orthologous OR proteins within the OR37 group across bee species. Sequences from broad polylectic species that show elevated ratios of nonsynonymous:synonymous substitutions in this protein are highlighted as in the previous figures. b) 3D structure of an OR subunit generated using a consensus sequence of aligned protein sequences within OR orthogroup 37 across bee species. Colors within the structure represent predicted local distance difference test (pLDDT) values and are a measure of model accuracy. Diversifying sites across the protein as inferred by FEL-CONTRAST models are mapped onto the structure as magenta spheres. Protein subunits identified within the structure are labeled according to previous structural models developed for OR genes and extracellular (ECL), transmembrane (TMB), and intracellular (ICL) domains are indicated. c) 3D structure shown in (b) with individual protein helices, labeled with transmembrane domain IDs, as per previously generated cryo-EM structures of OR proteins (del Mármol *et al*. 2021). Again, diversifying sites across the protein as inferred by FEL-CONTRAST models are mapped onto the structure as magenta spheres.

**Fig. 4.**
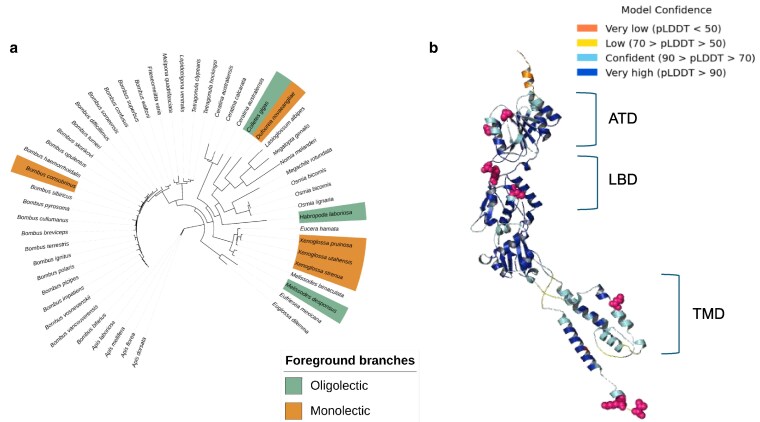
Gene tree and protein structure for IR orthogroup 9. a) Maximum likelihood phylogeny of orthologous IR proteins within the IR9 group across bee species. Sequences from monolectic and oligolectic species that show elevated ratios of nonsynonymous:synonymous substitutions in this protein are highlighted as in the previous figures. b) 3D structure of an IR subunit generated using a consensus sequence of aligned protein sequences within IR orthogroup 9 across bee species. Colors within the structure represent predicted local distance difference test (pLDDT) values and are a measure of model accuracy. Diversifying sites across the protein as inferred by FEL-CONTRAST models are mapped onto the structure as magenta spheres. Protein subunits identified within the structure are labeled according to previous structural models developed for IR genes and amino terminal domains (ATD), ligand-binding domains (LBD), and transmembrane domains (TMD) are indicated.

### Bees show higher rates of gene gains and losses in ORs compared to GRs and IRs

We modeled rates of gene turnover across ORs, GRs, and IRs through a birth/death model of gene family evolution, which posits that gene families evolve through processes of gene gain through random duplication and functionalization or loss through pseudogenization (Sánchez-Gracia *et al*. 2009). We used Computational Analysis of gene Family Evolution (CAFE v.5) (Mendes *et al*. 2020) to estimate λ, a parameter describing the probability of a given gene being lost or gained per unit of time across the phylogeny. We used λ rates for IRs as a point of comparison, as they are highly conserved across species within our dataset. We found the higher turnover rates in ORs (λ = 0.00485, -lnL = 2292.96, *E* = 0.0310, *k* = 3) than in GRs (λ = 0.00248, -lnL = 606.838, *E* = 0.0425, *k* = 3) comparable to those for IRs (λ = 0.00243, -lnL = 235.033, *E* = 0.0108, *k* = 2).

### Broad polylectic species have the highest rate of OR losses and GR gains compared to polylectic species

Next, to understand whether gene gain and loss rates differed across diet breadth categories for each gene family, we jointly estimated parameters for rates of gene gain ( ®β) and loss ( ®δ) for each species in the tree following differential equations described in Niimura *et al*. (2014) (Methods and Supplementary Table 11). We did not detect phylogenetic signal in estimated gain and loss rates for all three gene families (Supplementary Table 12). PGLS models were then fit to estimate whether gain and loss rates differed across diet breadth categories with BUSCO score as an explanatory variable for OR and IR models.

We recovered a significant relationship between OR loss rates and diet breadth (χ^2^ = 12.832, df = 3–47, *P* = 0.00501) such that loss rates were significantly higher in broad polylectic species compared to polylectic species (β = 0.00497, SE = 0.00141, *t* = 3.519, *P* = 0.00185) (Fig. 2c). Similarly, we found a significant relationship between GR gain rates and diet breadth (χ^2^ = 7.5388, df = 3–47, *P* = 0.05657) such that gain rates were significantly higher in broad polylectic species compared to polylectic species (β = 0.00359, SE = 0.001357, *t* = 2.645, *P* = 0.0349) (Fig. 2e). Gain and loss rates for IRs did not vary with diet breadth (IR gains: χ^2^ = 0.4367, df = 3–47, *P* = 0.9326; IR losses: χ^2^ = 4.8077, df = 3–47, *P* = 0.186) but IR loss rates did show a significant positive association with BUSCO scores (χ^2^ = 8.660, df = 1–47, *P* = 0.00325), congruent to our earlier finding that IR repertoires significantly varied with BUSCO scores (Supplementary Table 13).

We then fit a second set of PGLS models on a pruned dataset that corrected for the oversampling of species within the polylectic category within our dataset. This dataset contained 17 polylectic species, eight broad polylectic species, five monolectic species, and three oligolectic species. We again recovered a significant relationship between OR loss rates and diet breadth (χ^2^ = 8.533, df = 3–47, *P* = 0.0362) where broad polylectic species had significantly higher OR δ rates than polylectic species (β = 0.00452, SE = 0.00158, t = 2.861, *P* = 0.0195) (Supplementary Table 14). We also recovered a marginally significant relationship between GR gain rates and diet breadth (χ^2^ = 6.5620, df = 3–47, *P* = 0.0873), indicating that broad polylectic species showed higher gain rates than polylectic species (β = 0.00376, SE = 0.00153, *t* = 2.450, *P* = 0.0606). We found no relationship between IR δ rates and BUSCO scores (χ^2^ = 0.533, df = 1–47, *P* = 0.466).

### More olfactory receptor orthogroups show positive selection in monolectic than polylectic species

We combined results from branch-site unrestricted statistical tests for episodic diversification—phylogeny (BUSTED-Ph) and branch-site models implemented in PAML to identify eight sensory protein orthogroups putatively experiencing positive selection in four categories: within monolectic species, polylectic species, broad polylectic species, and combined monolectic and oligolectic species (Table 1 and Methods). Out of these, seven orthogroups across all three gene families (one IR orthogroup, three GR orthogroups, and three OR orthogroups) showed evidence of positive selection in specialist bees (monolectic species and species within both monolectic and oligolectic categories). We identified a single OR orthogroup that showed evidence of positive selection in broad polylectic species.

### 3D models of olfactory receptor genes reveal codon diversification across protein domains in specialist and generalist bees

The input amino acid alignments for all four OR orthogroups showed high levels of completeness (mean pairwise sequence identity was between 72% and 80%). Thus, we generated high-quality 3D structures representing each orthogroup comparable to the AbOrco reference structure (mean pLDDT scores were between 83% and 90.3% and TM-align scores ranged from 0.52 to 0.84) (Supplementary Table 18). Structural models for all OR orthogroups had the highest pLDDT scores of all the 3D models generated. Our structural models for OR orthogroups 3, 4, and 37 contained seven predicted transmembrane domains, as expected, while the model for OR orthogroup 24 contained only six transmembrane domains. We identified three diversifying codons within the structure of OR orthogroup 3, which contained OR67a-like genes and experienced positive selection in monolectic and oligolectic species. These codons occured within the extracellular portions of helix S4 and intracellular domains within helices S0 and S3. The structure of OR orthogroup 4, which contained putative orthologs to OR4a-like genes and showed diversification in monolectic and oligolectic species, contained a relatively long intracellular loop on the intracellular portion of helix S2 (residues 1–12) and five diversifying codons distributed in the intracellular regions of helix S0 and S3 and the extracellular portion of helix S5. The incomplete structure of OR orthogroup 24, which contain genes structurally similar to OR46a-like genes and experienced diversification in monolectic and oligolectic species, contained three codons identified by FEL-CONTRAST on the extracellular portions of helices S4 and S6 and transmembrane domains of helix S3. The structure of OR orthogroup 37, which contained putatively OR13a-like genes and showed positive selective pressures in broad polylectic species, contained eight diversifying codons within transmembrane domains of helices S2, S5, and S6 (Fig. 3, Table 1, Supplementary Tables 18 and 19, and Supplementary Fig. 5).

The codon alignment we used to construct the 3D model for IR orthogroup 9 showed diversification in specialists (monolectic and oligolectic species) and contains putative homologs to IR75a-like genes. However, this alignment had more gaps than for the ORs, which may reflect the much larger structure of these genes. Despite this, the structure we obtained was highly complete, with a mean pLDDT value of 86.3%. The crystal structure of insect IRs has not yet been identified. However, these receptors are highly conserved across phyla and are known to be structurally similar to AMPA subtype ionotropic glutamate receptors (Croset *et al*. 2010). We used this crystal structure as a comparison to ours and found that our IR9 model did not correspond closely to the AMPA iGluR structure (RMSD as calculated by PyMOL was 22.363 Å), potentially due to the additional extracellular amino-terminal domains found in vertebrate iGluRs as noted by previous studies (Rytz *et al*. 2013). We mapped nine diversifying sites onto this structure, five of which occurred in the extracellular ligand-binding domain and four identified within the intracellular portion of the ion channel pore (Ni, 2021; Wicher and Miazzi 2021) (Fig. 4, Table 1, and Supplementary Tables 18 and 19).

We further identified three orthologous groups of GR genes that showed diversification in specialist species, two of which diversified only in the most narrow category of specialization we used: monolectic species. The quality of the input alignment for GR orthogroup 1, which contained orthologs to GR64f proteins in bees and showed diversification in monolectic species, was comparable to the IR and OR alignments (mean sequence identity was 68%) and obtained a structural model containing seven transmembrane domains. Our structural model for this protein showed a single large intracellular loop between the S2 and S5 helices (residues 1–86) with low pLDDT scores (pLDDT < 50), which contained one codon experiencing diversification. The alignments for GR 7 and 8, which showed diversification in specialist bees and showed sequence similarity to genes within the GR28a/b cluster, were highly diverged, with mean sequence identities of 18% and 37%, respectively. The Alphafold structure for GR7 had only three transmembrane domains and showed overall low quality (70 < pLDDT < 90). We mapped a single diversifying codon onto this structure, which occurred within a potentially misfolded loop structure. The structural model for GR8 was of higher quality but was highly fragmented. We mapped four codons that showed diversification in monolectic species within putative transmembrane regions; our model did not allow us to identify helices showing diversification (Table 1, Supplementary Tables 18 and 19, and Supplementary Fig. 4).

## Discussion

We conducted the first large-scale analysis of olfactory receptor gene evolution within bees across dietary breadths, interrogating evolutionary patterns across gene and molecular scales. We base our analyses on orthologous groups of genes occurring across bee species, in order to ascertain broad patterns in olfactory gene evolution across bee diet breadths. Our findings suggest that specialization and generalization in bees involve distinct evolutionary patterns across both olfactory receptor gene families and individual genes. We found that broad generalist species tended to lose ORs and gain GRs faster than polylectic species and did not find associations between gain and loss rates of IR genes and dietary breadth in bees. We identified several orthologous groups of olfactory receptor genes showing evidence of accelerated amino acid substitutions within both specialist and generalist bees with more diversifying orthogroups occuring in specialists rather than generalists. Taken together, our results indicate that specialists are more likely to show changes in the molecular structure of receptors while generalists tend to lose OR and gain GR genes rapidly. Below, we situate our findings within what is known in other systems and identify new avenues of inquiry this study engenders.

Our estimated rates of gene turnover (as assessed by λ values) are similar to those reported for olfactory receptors in other insect systems (Sánchez-Gracia *et al*. 2009) suggesting that OR gain and loss rates are relatively rapid compared to those for GR and IRs in bees. This corroborates the findings of a previous study of OR gene evolution in corbiculate bees, which showed rapid lineage-specific expansions of ORs and high rates of diversification within paralogous genes (Brand and Ramírez 2017). OR genes are known to evolve through local tandem duplications and unequal crossing over events, leading to a conserved genomic structure where genes are present in large tandem arrays (Zhou *et al*. 2015; Brand and Ramírez 2017). Bees are known to be particularly dependent on olfactory cues for social communication, mating, and foraging (reviewed in Gomez Ramirez *et al*. 2023). OR evolution has been linked to complex social communication in eusocial honey bees (Mariette *et al*. 2021) and pheromone-mediated premating isolation in sympatric sister species of orchid bees (Brand *et al*. 2020). Conversely, we found that most of the GRs and IRs across bees were single-copy orthologs. The emergence of the earliest bees from Apoid wasps was accompanied by a significant reduction in GR repertoires (Robertson and Wanner 2006), leading to the hypothesis that bees may rely less on their sense of taste than other insects (Bestea *et al*. 2021). While GR turnover has been implicated in shifts in dietary breadth in other insect groups, particularly butterflies (Briscoe *et al*. 2013), little is known about whether GR turnover might underlie life history shifts and adaptation in bees.

Our estimates of variation in olfactory receptor gene gain and loss rates across bee diet breadths show that broad generalist (i.e., broad polylectic) species lose OR genes faster than other species. This result was robust to the bias toward polylectic species within our dataset. This study, to the best of our knowledge, is the first to examine relationships between diet breadth and olfactory gene evolution in a broad phylogenetic sample of bees. Our work complements the findings of a study reporting the recently assembled genome of the generalist pollinator *Tetragonisca angustula*, which shows that this species experienced a rapid loss in OR genes (Ferrari *et al*. 2024). However, our results contrast with studies on generalist foragers in other insect orders. Comparative studies on coleopteran (Mitchell *et al*. 2020) and lepidopteran (Engsontia *et al*. 2014) species have shown that generalist species tend to gain ORs faster than related specialists. For instance, in the case of the red flour beetle *Tribolium castaneum*, large gains in OR genes have been linked to the emergence of its broadly generalist diet (Engsontia *et al*. 2008).

The neural constraint hypothesis, which posits that constraints in the information processing ability of insects will curtail their ability to make efficient decisions in the recognition and acceptance of potential resources (Bernays 1998, 2001), can provide a framework to understand the observed loss of OR genes in broad generalist bees, which may indicate a relative loss of sensitivity in their olfactory systems. Generalist bees must be able to detect a broad range of floral resources and thus, often rely on floral volatile cues present across plant families during foraging (de Manincor *et al*. 2022). Thus, generalists may experience tradeoffs between their ability to detect a wide range of volatile chemicals and the precision of their ability to detect specific compounds, potentially due to the high metabolic costs of complex sensory system function (Levins and MacArthur 1969). This, in turn, may lead to generalists experiencing relaxed selection on sensory traits, leading to reductions in chemosensory or olfactory receptor diversity (Bernays and Wcislo 1994). This phenomenon has been demonstrated by behavioral experiments in herbivores, showing that the generalist species *Heliothis* responded less strongly to plant secondary compounds than a related specialist species (Bernays *et al*. 2000).

We complemented our exploration of olfactory receptor turnover rates by identifying orthologous genes diversifying across specialists and generalist bees, in order to determine potential molecular substrates driving diet breadth shifts. There has been substantial work on elucidating the function of tuning ORs in insects. For example, mutagenesis studies have established the importance of extracellular and transmembrane domains in helices S3, S5, S6, and S7 in both ligand-binding and modulating ion channel activity (Pacalon *et al*. 2023; Wang *et al*. 2024 ; Zhao *et al*. 2024). The function and structure of IRs in insects is a newly emerging field and, while there is high variation in sequence structure across IR proteins, ligand-binding domains and pore channel domains have been identified across IRs in *D. melanogaster* (Ni, 2021). Further, point mutations within both regions impact the functionality of IR channels, indicating that conformation changes across the protein complex are involved in activation of IRs (Abuin *et al*. 2019). We identified OR and IR genes that showed diversification within the relevant protein domains within specialists and generalists, potentially indicating shifts in ligand-binding affinity and tuning breadths of these genes that might facilitate shifts in diet breadth. GRs are structurally similar to ORs, reflecting their shared evolutionary history, and ligand-binding involves extracellular pockets within helices S5, S6, and S7 (Ma *et al*. 2024). However, the low quality of our 3D models precludes our ability to hypothesize on the functional consequences of diversifying codons within GRs in specialists.

Functional information in bees for the receptor orthogroups identified as experiencing positive selection in our dataset were largely absent, as few studies to date have characterized ligands for these receptors in bee species. While functional information is available for orthologous proteins within the fly *D. melanogaster*, the large divergence between bee and fly olfactory receptors limits our ability to draw conclusions on ligand-binding specificity in bees (Andersson *et al*. 2015). We were, however, able to find functional information in bees for the GR orthogroup GR1, which contained orthologs to AmGR1sequences in *A. mellifera*, which show homology with GR64f sequences in *D. melanogaster*. This gene is a conserved coreceptor for GR complexes that respond to sugars such as sucrose, maltose, and trehalose (Jiao *et al*. 2008) and is known to be conserved across insect orders (Kent and Robertson 2009). Codon-based branch-site tests are highly conservative and prone to false negatives in genes with high rates of synonymous codon substitutions, thus the number of positively selected genes identified here is likely conservative (Gharib and Robinson-Rechavi 2013). However, since the sample size for specialist species within our dataset is small, the conservative nature of these tests allows us to identify a preliminary set of genes that may be fruitful targets for future functional and physiological studies.

These findings on specialist bees provide a contrast to our findings on generalists, which show higher rates of OR gene loss that may be attributed to relaxed selective pressures on olfactory sensory genes, leading to higher rates of gene loss and dampened olfactory capabilities. Positive selective pressures on olfactory receptor genes in specialists and the resulting diversification of their odorant binding properties may be due to specialists being less constrained by tradeoffs in the complexity and specificity of neural systems and thus allocating neural resources toward enhancing compound detection (Futuyma and Moreno 1988). Olfactory sensory receptors are key components of the peripheral olfactory systems of insects and are the first links between insect brains and their environments (Andersson *et al*. 2015). As such, these peripheral systems are known to be highly responsive to environmental and ecological pressures faced by insects and their evolution is linked to shifts in higher-order processing within the cerebral ganglion (Hansson and Stensmyr 2011).

Since we were able to identify individual orthologous groups of genes that showed divergence across divergent specialist species, our results also support a paradigm of specialization via parallel adaptive divergence of specific sensory proteins across specialists (Martin *et al*. 2011). The diversity of behaviors found across insects despite a relative homology of neuromodulatory systems across taxa has been linked to conserved genetic and neuronal substrates that are coopted during adaptation to similar ecological challenges across species (Katz and Lillvis 2014; Vertacnik and Linnen 2017). Studies within the group *Drosophila* have identified that changes in the tuning of a single sensory neuron, ab3A, facilitate attraction to oviposition substrates across species; it responds to host-specific cues in the specialists *D. sechellia* (oviposits on Noni), *D. melanogaster* (oviposits on Marula), and *D. suzukii* (oviposits on ripe fruit) (Zhao and McBride 2020). In sweat bees, a comparative study across social and nonsocial species implicated diversification in genes involved in juvenile hormone transportation in the evolution of eusociality, pointing to the cooption of an ancestral endocrine pathway during transitions to group living (Jones *et al*. 2023). The chemosensory genes identified through this study are potentially fruitful candidates to investigate the genetic architecture of pollen specialization across bee clades.

Our results on chemosensory receptor evolution across bees should be interpreted in the light of several caveats. Crucially, dietary breadth in bees covaries with other life history traits that are also linked to the evolution of sensory systems, particularly sociality. Social bees are often generalists, as they have to forage for enough resources to support large colonies and these colonies survive over much longer periods than solitary bees, both necessitating foraging on a wide range of available plant resources (Wood *et al*. 2023). Alongside, previous work has shown that eusocial bees tend to experience rapid expansions in OR repertoires (Zhou *et al*. 2015). This has been linked to the need for communication between individuals within a colony, which is often mediated by cuticular hydrocarbons and pheromone compounds and thus, requires specific olfactory channels to process this information (Couto *et al*. 2023). Indeed, we find that OR orthogroup 4, which showed signatures of positive selection in the specialists in our dataset, was orthologous to receptors known to be involved in social communication. In *A. mellifera*, this receptor is a candidate receptor for fatty acids and may be involved in detecting queen mandibular gland secretions, aiding chemical communication between castes (Karpe *et al*. 2017). While our dataset does not allow us to disentangle the effects of sociality and pollen specialization on diet breadth, our sampling includes both solitary and social specialists within the “polylectic” and “broad polylectic” categories. Future work might focus on comparisons of specialists and generalists across varying levels of sociality.

A further caveat is that pollen diet breadth is often difficult to define without direct evidence of pollen collection (such as behavioral observations and identification of pollen sources from bee scopal hairs) by female bees. Bees often collect nectar from multiple plant species while restricting the diversity of pollen they collect. Further, individual bees may show large variation in the diversity of pollen resources they use (Cane and Sipes 2006). The depth of inquiry across species within our dataset varies widely and thus, we used a conservative approach to assign diet breadth as categorical variables in order to collapse the aforementioned sources of variation within diet breadths. However, this approach reduced the sample size of specialists within our dataset, reducing the statistical power. Therefore, while our dataset indicates that oligolectic bees showed increased OR repertoires and decreased IR repertoires, this result must be interpreted with caution given the low sample size within this category (*N* = 3).

A growing consensus in the field of insect genomics is the need to broaden sampling to represent insect diversity more fully. Our dataset is heavily biased toward species within the family Apidae, which is a large, cosmopolitan group containing most of the commonly studied and managed bee species (i.e., species within the genera *Apis* and *Bombus*). As these species tend to be eusocial and generalists, our sampling reflects a bias toward generalist bee palynivores in the species that have been sequenced thus far. Other bee families that are not included in our sample, such as Melittidae and Andrenidae, are dominated by specialist species. The insect i5k project (i5K Consortium 2013) and the Beenome100 initiative (https://www.beenome100.org/) are already providing a broad set of insect genomes that can be useful for further explorations of trait variation across bees and other insects. We hope that these results can lead to further evolutionary and functional investigations of bee olfactory receptors, with a focus on less-studied specialist species. This system provides an intriguing contrast to other well-studied systems and presents new insights into how dietary niche occupancy evolves across taxa.

## Supplementary Material

jkaf105_Supplementary_Data

## Data Availability

WGS and antennal RNAseq datasets generated for this project have been deposited into the SRA in the National Centre for Biotechnology Information database (Bioproject accession PRJNA1095984). Pipelines used for bioinformatic and statistical analyses are available at https://github.com/Avehi/bee-olfactory-receptors. Raw data files (including alignments, trees, gene turnover analyses, and molecular evolution analyses) are available on the figshare database at https://doi.org/10.6084/m9.figshare.28598129.v1. Supplemental material available at G3 online.

## References

[jkaf105-B1] Abuin L, Prieto-Godino LL, Pan H, Gutierrez C, Huang L, Jin R, Benton R. 2019. In vivo assembly and trafficking of olfactory ionotropic receptors. BMC Biol. 17(1):34. doi:10.1186/s12915-019-0651-7. 30995910 PMC6472016

[jkaf105-B2] Almeida EAB, Bossert S, Danforth BN, Porto DS, Freitas FV, Davis CC, Murray EA, Blaimer BB, Spasojevic T, Ströher PR, et al 2023. The evolutionary history of bees in time and space. Curr Biol. 33(16):3409–3422.e6. doi:10.1016/j.cub.2023.07.005. 37506702

[jkaf105-B3] Alonge M, Lebeigle L, Kirsche M, Jenike K, Ou S, Aganezov S, Wang X, Lippman ZB, Schatz MC, Soyk S. 2022. Automated assembly scaffolding using RagTag elevates a new tomato system for high-throughput genome editing. Genome Biol. 23(1):258. doi:10.1186/s13059-022-02823-7. 36522651 PMC9753292

[jkaf105-B4] Álvarez-Carretero S, Kapli P, Yang Z. 2023. Beginner's guide on the use of PAML to detect positive selection. Mol Biol Evol. 40(4):msad041. doi:10.1093/molbev/msad041. 37096789 PMC10127084

[jkaf105-B5] Andersson MN, Löfstedt C, Newcomb RD. 2015. Insect olfaction and the evolution of receptor tuning. Front Ecol Evol. 3:53. doi:10.3389/fevo.2015.00053.

[jkaf105-B6] Armstrong J, Hickey G, Diekhans M, Fiddes IT, Novak AM, Deran A, Fang Q, Xie D, Feng S, Stiller J, et al 2020. Progressive Cactus is a multiple-genome aligner for the thousand-genome era. Nature. 587(7833):246–251. doi:10.1038/s41586-020-2871-y. 33177663 PMC7673649

[jkaf105-B7] Auer TO, Khallaf MA, Silbering AF, Zappia G, Ellis K, Álvarez-Ocaña R, Arguello JR, Hansson BS, Jefferis GSXE, Caron SJC, et al 2020. Olfactory receptor and circuit evolution promote host specialization. Nature. 579(7799):402–408. doi:10.1038/s41586-020-2073-7. 32132713 PMC7100913

[jkaf105-B8] Auer TO, Shahandeh MP, Benton R. 2021. *Drosophila sechellia*: a genetic model for behavioral evolution and neuroecology. Annu Rev Genet. 55(1):527–554. 10.1146/annurev-genet-071719-020719. 34530638

[jkaf105-B9] Bernays EA . 1998. Evolution of feeding behavior in insect herbivores. BioScience. 48(1):35–44. doi:10.2307/1313226.

[jkaf105-B10] Bernays EA . 2001. Neural limitations in phytophagous insects: implications for diet breadth and evolution of host affiliation. Annu Rev Entomol. 46(1):703–727. doi:10.1146/annurev.ento.46.1.703. 11112184

[jkaf105-B11] Bernays EA, Oppenheim S, Chapman RF, Kwon H, Gould F. 2000. Taste sensitivity of insect herbivores to deterrents is greater in specialists than in generalists: a behavioral test of the hypothesis with two closely related caterpillars. J Chem Ecol. 26(2):547–563. doi:10.1023/A:1005430010314.

[jkaf105-B12] Bernays EA, Wcislo WT. 1994. Sensory capabilities, information processing, and resource specialization. Q Rev Biol. 69(2):187–204. doi:10.1086/418539.

[jkaf105-B13] Bestea L, Réjaud A, Sandoz J-C, Carcaud J, Giurfa M, de Brito Sanchez MG. 2021. Peripheral taste detection in honey bees: what do taste receptors respond to? Eur J Neurosci. 54(2):4417–4444. doi:10.1111/ejn.15265. 33934411

[jkaf105-B14] Bianchini G, Sánchez-Baracaldo P. 2024. TreeViewer: flexible, modular software to visualise and manipulate phylogenetic trees. Ecol Evol. 14(2):e10873. doi:10.1002/ece3.10873. 38314311 PMC10834882

[jkaf105-B15] Bohbot JD, Pitts RJ. 2015. The narrowing olfactory landscape of insect odorant receptors. Front Ecol Evol. 3:39. doi:10.3389/fevo.2015.00039.

[jkaf105-B16] Bohbot J, Pitts RJ, Kwon H-W, Rützler M, Robertson HM, Zwiebel LJ. 2007. Molecular characterization of the *Aedes aegypti* odorant receptor gene family. Insect Mol Biol. 16(5):525–537. 10.1111/j.1365-2583.2007.00748.x. 17635615 PMC3100214

[jkaf105-B17] Bolger AM, Lohse M, Usadel B. 2014. Trimmomatic: a flexible trimmer for Illumina sequence data. Bioinformatics. 30(15):2114–2120. doi:10.1093/bioinformatics/btu170. 24695404 PMC4103590

[jkaf105-B18] Brand P, Hinojosa-Díaz IA, Ayala R, Daigle M, Yurrita Obiols CL, Eltz T, Ramírez SR. 2020. The evolution of sexual signaling is linked to odorant receptor tuning in perfume-collecting orchid bees. Nat Commun. 11(1):244. doi:10.1038/s41467-019-14162-6. 31932598 PMC6957680

[jkaf105-B19] Brand P, Ramírez SR. 2017. The evolutionary dynamics of the odorant receptor gene family in corbiculate bees. Genome Biol Evol. 9(8):2023–2036. doi:10.1093/gbe/evx149. 28854688 PMC5597890

[jkaf105-B20] Brand P, Robertson HM, Lin W, Pothula R, Klingeman WE, Jurat-Fuentes JL, Johnson BR. 2018. The origin of the odorant receptor gene family in insects. Elife. 7:e38340. 10.7554/eLife.38340. 30063003 PMC6080948

[jkaf105-B21] Branstetter MG, Danforth BN, Pitts JP, Faircloth BC, Ward PS, Buffington ML, Gates MW, Kula RR, Brady SG. 2017. Phylogenomic insights into the evolution of stinging wasps and the origins of ants and bees. Curr Biol. 27(7):1019–1025. doi:10.1016/j.cub.2017.03.027. 28376325

[jkaf105-B22] Briscoe AD, Macias-Muñoz A, Kozak KM, Walters JR, Yuan F, Jamie GA, Martin SH, Dasmahapatra KK, Ferguson LC, Mallet J, et al 2013. Female behaviour drives expression and evolution of gustatory receptors in butterflies. PLoS Genet. 9(7):e1003620. doi:10.1371/journal.pgen.1003620. 23950722 PMC3732137

[jkaf105-B23] Bryant P, Pozzati G, Elofsson A. 2022. Improved prediction of protein-protein interactions using AlphaFold2. Nat Commun. 13(1):1265. doi:10.1038/s41467-022-28865-w. 35273146 PMC8913741

[jkaf105-B24] Bushnell B, Rood J, Singer E. 2017. BBMerge—accurate paired shotgun read merging via overlap. PLoS One. 12(10):e0185056. doi:10.1371/journal.pone.0185056. 29073143 PMC5657622

[jkaf105-B25] Butterwick JA, del Mármol J, Kim KH, Kahlson MA, Rogow JA, Walz T, Ruta V. 2018. Cryo-EM structure of the insect olfactory receptor Orco. Nature. 560(7719):447–452. doi:10.1038/s41586-018-0420-8. 30111839 PMC6129982

[jkaf105-B26] Cane JH . 2021. A brief review of monolecty in bees and benefits of a broadened definition. Apidologie. 52(1):17–22. doi:10.1007/s13592-020-00785-y.

[jkaf105-B27] Cane JH, Sipes S. 2006. Floral specialization by bees: analytical methods and a revised lexicon for oligolecty. In: Waser N M, Ollerton J, editors. Plant-pollinator interactions: from specialization to generalization. University of Chicago Press. p. 99–122.

[jkaf105-B28] Capella-Gutiérrez S, Silla-Martínez JM, Gabaldón T. 2009. Trimal: a tool for automated alignment trimming in large-scale phylogenetic analyses. Bioinformatics. 25(15):1972–1973. doi:10.1093/bioinformatics/btp348. 19505945 PMC2712344

[jkaf105-B29] Cardinal S, Danforth BN. 2013. Bees diversified in the age of eudicots. Proc Biol Sci. 280(1755):20122686. doi:10.1098/rspb.2012.2686. 23363629 PMC3574388

[jkaf105-B30] Chen N . 2004. Using RepeatMasker to identify repetitive elements in genomic sequences. Curr Protoc Bioinformatics. Chapter 4(1):Unit 4. doi:10.1002/0471250953.bi0410s05. 18428725

[jkaf105-B31] Couto A, Marty S, Dawson EH, d’Ettorre P, Sandoz J-C, Montgomery SH. 2023. Evolution of the neuronal substrate for kin recognition in social Hymenoptera. Biol Rev Camb Philos Soc. 98(6):2226–2242. doi:10.1111/brv.13003. 37528574

[jkaf105-B32] Croset V, Rytz R, Cummins SF, Budd A, Brawand D, Kaessmann H, Gibson TJ, Benton R. 2010. Ancient protostome origin of chemosensory ionotropic glutamate receptors and the evolution of insect taste and olfaction. PLoS Genet. 6(8):e1001064. doi:10.1371/journal.pgen.1001064. 20808886 PMC2924276

[jkaf105-B33] del Mármol J, Yedlin MA, Ruta V. 2021. The structural basis of odorant recognition in insect olfactory receptors. Nature. 597(7874):126–131. doi:10.1038/s41586-021-03794-8. 34349260 PMC8410599

[jkaf105-B34] de Manincor N, Andreu B, Buatois B, Lou Chao H, Hautekèete N, Massol F, Piquot Y, Schatz B, Schmitt E, Dufay M. 2022. Geographical variation of floral scents in generalist entomophilous species with variable pollinator communities. Funct Ecol. 36(3):763–778. doi:10.1111/1365-2435.13984.

[jkaf105-B35] Dhakal S, Sang J, Aryal B, Lee Y. 2021. Ionotropic receptors mediate nitrogenous waste avoidance in *Drosophila melanogaster*. Commun Biol. 4(1):1281. doi:10.1038/s42003-021-02799-3. 34773080 PMC8589963

[jkaf105-B36] Dobin A, Davis CA, Schlesinger F, Drenkow J, Zaleski C, Jha S, Batut P, Chaisson M, Gingeras TR. 2013. STAR: ultrafast universal RNA-seq aligner. Bioinformatics. 29(1):15–21. doi:10.1093/bioinformatics/bts635. 23104886 PMC3530905

[jkaf105-B37] Dorchin A, Shafir A, Neumann FH, Langgut D, Vereecken NJ, Mayrose I. 2021. Bee flowers drive macroevolutionary diversification in long-horned bees. Proc Biol Sci. 288(1959):20210533. doi:10.1098/rspb.2021.0533. 34547912 PMC8515878

[jkaf105-B38] Engsontia P, Sanderson AP, Cobb M, Walden KKO, Robertson HM, Brown S. 2008. The red flour beetle's large nose: an expanded odorant receptor gene family in *Tribolium castaneum*. Insect Biochem Mol Biol. 38(4):387–397. doi:10.1016/j.ibmb.2007.10.005. 18342245

[jkaf105-B39] Engsontia P, Sangket U, Chotigeat W, Satasook C. 2014. Molecular evolution of the odorant and gustatory receptor genes in lepidopteran insects: implications for their adaptation and speciation. J Mol Evol. 79(1–2):21–39. doi:10.1007/s00239-014-9633-0. 25038840

[jkaf105-B40] Ferrari RR, Ricardo PC, Dias FC, de Souza Araujo N, Soares DO, Zhou Q-S, Zhu C-D, Coutinho LL, Arias MC, Batista TM. 2024. The nuclear and mitochondrial genome assemblies of *Tetragonisca angustula* (Apidae: Meliponini), a tiny yet remarkable pollinator in the Neotropics. BMC Genomics. 25(1):587. doi:10.1186/s12864-024-10502-z. 38862915 PMC11167848

[jkaf105-B41] Fiddes IT, Armstrong J, Diekhans M, Nachtweide S, Kronenberg ZN, Underwood JG, Gordon D, Earl D, Keane T, Eichler EE, et al 2018. Comparative Annotation Toolkit (CAT)—simultaneous clade and personal genome annotation. Genome Res. 28(7):1029–1038. doi:10.1101/gr.233460.117. 29884752 PMC6028123

[jkaf105-B42] Forêt S, Maleszka R. 2006. Function and evolution of a gene family encoding odorant binding-like proteins in a social insect, the honey bee (*Apis mellifera*). Genome Res. 16(11):1404–1413. doi:10.1101/gr.5075706. 17065610 PMC1626642

[jkaf105-B43] Fox J, Weisberg S. 2019. An R Companion to Applied Regression. Third edition. Thousand Oaks (CA): Sage. https://www.john-fox.ca/Companion/.

[jkaf105-B44] Futuyma DJ, Moreno G. 1988. The evolution of ecological specialization. Annu Rev Ecol Syst. 19(1):207–233. doi:10.1146/annurev.es.19.110188.001231.

[jkaf105-B45] Gharib WH, Robinson-Rechavi M. 2013. The branch-site test of positive selection is surprisingly robust but lacks power under synonymous substitution saturation and variation in GC. Mol Biol Evol. 30(7):1675–1686. doi:10.1093/molbev/mst062. 23558341 PMC3684852

[jkaf105-B46] Girgis HZ . 2015. Red: an intelligent, rapid, accurate tool for detecting repeats de-novo on the genomic scale. BMC Bioinformatics. 16(1):227. doi:10.1186/s12859-015-0654-5. 26206263 PMC4513396

[jkaf105-B47] Goldman-Huertas B, Mitchell RF, Lapoint RT, Faucher CP, Hildebrand JG, Whiteman NK. 2015. Evolution of herbivory in Drosophilidae linked to loss of behaviors, antennal responses, odorant receptors, and ancestral diet. Proc Natl Acad Sci U S A. 112(10):3026–3031. doi:10.1073/pnas.1424656112. 25624509 PMC4364187

[jkaf105-B48] Gomes JV, Singh-Bhagania S, Cenci M, Chacon Cordon C, Singh M, Butterwick JA. 2024. The molecular basis of sugar detection by an insect taste receptor. Nature. 629(8010):228–234. doi:10.1038/s41586-024-07255-w. 38447670 PMC11062906

[jkaf105-B49] Gomez Ramirez WC, Thomas NK, Muktar IJ, Riabinina O. 2023. The neuroecology of olfaction in bees. Curr Opin Insect Sci. 56:101018. doi:10.1016/j.cois.2023.101018. 36842606

[jkaf105-B50] Gurevich A, Saveliev V, Vyahhi N, Tesler G. 2013. QUAST: quality assessment tool for genome assemblies. Bioinformatics. 29(8):1072–1075. doi:10.1093/bioinformatics/btt086. 23422339 PMC3624806

[jkaf105-B51] Hansson BS, Stensmyr MC. 2011. Evolution of insect olfaction. Neuron. 72(5):698–711. doi:10.1016/j.neuron.2011.11.003. 22153368

[jkaf105-B52] Hothorn T, Bretz F, Westfall P. 2008. Simultaneous inference in general parametric models. Biom J. 50(3):346–363. doi:10.1002/bimj.200810425. 18481363

[jkaf105-B53] Howell AD, Alarcón R. 2007. Osmia bees (Hymenoptera: Megachilidae) can detect nectar-rewarding flowers using olfactory cues. Anim Behav. 74(2):199–205. doi:10.1016/j.anbehav.2006.11.012.

[jkaf105-B54] Hu J, Fan J, Sun Z, Liu S. 2020. NextPolish: a fast and efficient genome polishing tool for long-read assembly. Bioinformatics. 36(7):2253–2255. doi:10.1093/bioinformatics/btz891. 31778144

[jkaf105-B55] i5K Consortium . 2013. The i5K initiative: advancing arthropod genomics for knowledge, human health, agriculture, and the environment. J Hered. 104(5):595–600. doi:10.1093/jhered/est050. 23940263 PMC4046820

[jkaf105-B56] Jiao Y, Moon SJ, Wang X, Ren Q, Montell C. 2008. Gr64f is required in combination with other gustatory receptors for sugar detection in *Drosophila*. Curr Biol. 18(22):1797–1801. doi:10.1016/j.cub.2008.10.009. 19026541 PMC2676565

[jkaf105-B57] Jones BM, Rubin BER, Dudchenko O, Kingwell CJ, Traniello IM, Wang ZY, Kapheim KM, Wyman ES, Adastra PA, Liu W, et al 2023. Convergent and complementary selection shaped gains and losses of eusociality in sweat bees. Nat Ecol Evol. 7(4):557–569. doi:10.1038/s41559-023-02001-3. 36941345 PMC11610481

[jkaf105-B58] Kalyaanamoorthy S, Minh BQ, Wong TKF, von Haeseler A, Jermiin LS. 2017. ModelFinder: fast model selection for accurate phylogenetic estimates. Nat Methods. 14(6):587–589. doi:10.1038/nmeth.4285. 28481363 PMC5453245

[jkaf105-B59] Karpe SD, Dhingra S, Brockmann A, Sowdhamini R. 2017. Computational genome-wide survey of odorant receptors from two solitary bees *Dufourea novaeangliae* (Hymenoptera: Halictidae) and *Habropoda laboriosa* (Hymenoptera: Apidae). Sci Rep. 7(1):10823. doi:10.1038/s41598-017-11098-z. 28883425 PMC5589748

[jkaf105-B60] Katoh K, Standley DM. 2013. MAFFT multiple sequence alignment software version 7: improvements in performance and usability. Mol Biol Evol. 30(4):772–780. doi:10.1093/molbev/mst010. 23329690 PMC3603318

[jkaf105-B61] Katz PS, Lillvis JL. 2014. Reconciling the deep homology of neuromodulation with the evolution of behavior. Curr Opin Neurobiol. 29:39–47. doi:10.1016/j.conb.2014.05.002. 24878891

[jkaf105-B62] Kent LB, Robertson HM. 2009. Evolution of the sugar receptors in insects. BMC Evol Biol. 9(1):41. doi:10.1186/1471-2148-9-41. 19226470 PMC2667405

[jkaf105-B63] Kosakovsky Pond SL, Poon AFY, Velazquez R, Weaver S, Hepler NL, Murrell B, Shank SD, Magalis BR, Bouvier D, Nekrutenko A, et al 2020. Hyphy 2.5—a customizable platform for evolutionary hypothesis testing using phylogenies. Mol Biol Evol. 37(1):295–299. doi:10.1093/molbev/msz197. 31504749 PMC8204705

[jkaf105-B64] Kosakovsky Pond SL, Wisotsky SR, Escalante A, Magalis BR, Weaver S. 2021. Contrast-FEL—a test for differences in selective pressures at individual sites among clades and sets of branches. Mol Biol Evol. 38(3):1184–1198. doi:10.1093/molbev/msaa263. 33064823 PMC7947784

[jkaf105-B65] Krogh A, Larsson B, von Heijne G, Sonnhammer EL. 2001. Predicting transmembrane protein topology with a hidden Markov model: application to complete genomes. J Mol Biol. 305(3):567–580. doi:10.1006/jmbi.2000.4315. 11152613

[jkaf105-B66] Leinonen R, Sugawara H, Shumway M. 2011. The sequence read archive. Nucleic Acids Res. 39(Database Issue):D19–21. 10.1093/nar/gkq1019. 21062823 PMC3013647

[jkaf105-B67] Letunic I, Bork P. 2021. Interactive Tree Of Life (iTOL) v5: an online tool for phylogenetic tree display and annotation. Nucleic Acids Res. 49(W1):W293–W296. doi:10.1093/nar/gkab301. 33885785 PMC8265157

[jkaf105-B68] Levins R, MacArthur R. 1969. An hypothesis to explain the incidence of monophagy. Ecology. 50(5):910–911. doi:10.2307/1933709.

[jkaf105-B69] Levy Karin E, Mirdita M, Söding J. 2020. MetaEuk—sensitive, high-throughput gene discovery, and annotation for large-scale eukaryotic metagenomics. Microbiome. 8(1):48. doi:10.1186/s40168-020-00808-x. 32245390 PMC7126354

[jkaf105-B70] Li H . 2016. Minimap and miniasm: fast mapping and de novo assembly for noisy long sequences. Bioinformatics. 32(14):2103–2110. doi:10.1093/bioinformatics/btw152. 27153593 PMC4937194

[jkaf105-B71] Li H, Handsaker B, Wysoker A, Fennell T, Ruan J, Homer N, Marth G, Abecasis G, Durbin R, 1000 Genome Project Data Processing Subgroup. 2009. The sequence alignment/map format and SAMtools. Bioinformatics. 25(16):2078–2079. doi:10.1093/bioinformatics/btp352. 19505943 PMC2723002

[jkaf105-B72] Ma D, Hu M, Yang X, Liu Q, Ye F, Cai W, Wang Y, Xu X, Chang S, Wang R, et al 2024. Structural basis for sugar perception by *Drosophila* gustatory receptors. Science. 383(6685):eadj2609. doi:10.1126/science.adj2609. 38305684

[jkaf105-B73] Manni M, Berkeley MR, Seppey M, Simao FA, Zdobnov EM. 2021. BUSCO update: novel and streamlined workflows along with broader and deeper phylogenetic coverage for scoring of eukaryotic, prokaryotic, and viral genomes. Mol Biol Evol. 38(10):4647–4654. doi:10.1093/molbev/msab199. 34320186 PMC8476166

[jkaf105-B74] Mariette J, Carcaud J, Sandoz J-C. 2021. The neuroethology of olfactory sex communication in the honeybee *Apis mellifera* L. Cell Tissue Res. 383(1):177–194. doi:10.1007/s00441-020-03401-8. 33447877

[jkaf105-B75] Martin JP, Beyerlein A, Dacks AM, Reisenman CE, Riffell JA, Lei H, Hildebrand JG. 2011. The neurobiology of insect olfaction: sensory processing in a comparative context. Prog Neurobiol. 95(3):427–447. doi:10.1016/j.pneurobio.2011.09.007. 21963552

[jkaf105-B76] Matsunaga T, Reisenman CE, Goldman-Huertas B, Brand P, Miao K, Suzuki HC, Verster KI, Ramírez SR, Whiteman NK. 2022. Evolution of olfactory receptors tuned to mustard oils in herbivorous Drosophilidae. Mol Biol Evol. 39(2):msab362. doi:10.1093/molbev/msab362. 34963012 PMC8826531

[jkaf105-B77] Mendes FK, Vanderpool D, Fulton B, Hahn MW. 2020. CAFE 5 models variation in evolutionary rates among gene families. Bioinformatics. 36(22–23):5516–5518. doi:10.1093/bioinformatics/btaa1022. 33325502

[jkaf105-B78] Minckley RL, Cane JH, Kervin L. 2000. Origins and ecological consequences of pollen specialization among desert bees. Proc Biol Sci. 267(1440):265–271. doi:10.1098/rspb.2000.0996. 10714881 PMC1690526

[jkaf105-B79] Minh BQ, Schmidt HA, Chernomor O, Schrempf D, Woodhams MD, von Haeseler A, Lanfear R. 2020. IQ-TREE 2: new models and efficient methods for phylogenetic inference in the genomic era. Mol Biol Evol. 37(5):1530–1534. doi:10.1093/molbev/msaa015. 32011700 PMC7182206

[jkaf105-B80] Mirarab S, Reaz R, Bayzid MS, Zimmermann T, Swenson MS, Warnow T. 2014. ASTRAL: genome-scale coalescent-based species tree estimation. Bioinformatics. 30(17):i541–i548. doi:10.1093/bioinformatics/btu462. 25161245 PMC4147915

[jkaf105-B81] Mirdita M, Schütze K, Moriwaki Y, Heo L, Ovchinnikov S, Steinegger M. 2022. ColabFold: making protein folding accessible to all. Nat Methods. 19(6):679–682. doi:10.1038/s41592-022-01488-1. 35637307 PMC9184281

[jkaf105-B82] Mitchell RF, Schneider TM, Schwartz AM, Andersson MN, McKenna DD. 2020. The diversity and evolution of odorant receptors in beetles (Coleoptera). Insect Mol Biol. 29(1):77–91. doi:10.1111/imb.12611. 31381201

[jkaf105-B83] Murrell B, Weaver S, Smith MD, Wertheim JO, Murrell S, Aylward A, Eren K, Pollner T, Martin DP, Smith DM, et al 2015. Gene-wide identification of episodic selection. Mol Biol Evol. 32(5):1365–1371. doi:10.1093/molbev/msv035. 25701167 PMC4408417

[jkaf105-B84] Nachtweide S, Stanke M. 2019. Multi-genome annotation with AUGUSTUS. In: Kollmar M, editors. Gene prediction: methods and protocols. Humana New York, NY: Springer. p. 139–160. doi:10.1007/978-1-4939-9173-0_8. 31020558

[jkaf105-B85] Ngoc PCT, Greenhalgh R, Dermauw W, Rombauts S, Bajda S, Zhurov V, Grbić M, Van de Peer Y, Van Leeuwen T, Rouzé P, et al 2016. Complex evolutionary dynamics of massively expanded chemosensory receptor families in an extreme generalist chelicerate herbivore. Genome Biol Evol. 8(11):3323–3339. doi:10.1093/gbe/evw249. 27797949 PMC5203786

[jkaf105-B86] Ni L . 2021. The structure and function of ionotropic receptors in *Drosophila*. Front Mol Neurosci. 13:638839. doi:10.3389/fnmol.2020.638839. 33597847 PMC7882480

[jkaf105-B87] Niimura Y, Matsui A, Touhara K. 2014. Extreme expansion of the olfactory receptor gene repertoire in African elephants and evolutionary dynamics of orthologous gene groups in 13 placental mammals. Genome Res. 24(9):1485–1496. doi:10.1101/gr.169532.113. 25053675 PMC4158756

[jkaf105-B88] Obiero GF, Pauli T, Geuverink E, Veenendaal R, Niehuis O, Große-Wilde E. 2021. Chemoreceptor diversity in apoid wasps and its reduction during the evolution of the pollen-collecting lifestyle of bees (Hymenoptera: Apoidea). Genome Biol Evol. 13(3):evaa269. doi:10.1093/gbe/evaa269. 33484563 PMC8011036

[jkaf105-B89] Pacalon J, Audic G, Magnat J, Philip M, Golebiowski J, Moreau CJ, Topin J. 2023. Elucidation of the structural basis for ligand binding and translocation in conserved insect odorant receptor co-receptors. Nat Commun. 14(1):8182. doi:10.1038/s41467-023-44058-5. 38081900 PMC10713630

[jkaf105-B90] Paradis E, Schliep K. 2019. Ape 5.0: an environment for modern phylogenetics and evolutionary analyses in R. Bioinformatics. 35(3):526–528. doi:10.1093/bioinformatics/bty633. 30016406

[jkaf105-B91] Paulino D, Warren RL, Vandervalk BP, Raymond A, Jackman SD, Birol I. 2015. Sealer: a scalable gap-closing application for finishing draft genomes. BMC Bioinformatics. 16(1):230. doi:10.1186/s12859-015-0663-4. 26209068 PMC4515008

[jkaf105-B92] Peters RS, Krogmann L, Mayer C, Donath A, Gunkel S, Meusemann K, Kozlov A, Podsiadlowski L, Petersen M, Lanfear R, et al 2017. Evolutionary history of the hymenoptera. Curr Biol. 27(7):1013–1018. doi:10.1016/j.cub.2017.01.027. 28343967

[jkaf105-B93] Pinheiro J, Bates D; R Core Team. 2025. nlme: Linear and Nonlinear Mixed Effects Models. R package version 3.1-168 [accessed 2025 Mar 31]. https://CRAN.R-project.org/package=nlme.

[jkaf105-B94] Pope NS, Singh A, Childers AK, Kapheim KM, Evans JD, López-Uribe MM. 2023. The expansion of agriculture has shaped the recent evolutionary history of a specialized squash pollinator. Proc Natl Acad Sci U S A. 120(15):e2208116120. doi:10.1073/pnas.2208116120. 37011184 PMC10104555

[jkaf105-B95] Prjibelski A, Antipov D, Meleshko D, Lapidus A, Korobeynikov A. 2020. Using SPAdes de novo assembler. Curr Protoc Bioinformatics. 70(1):e102. doi:10.1002/cpbi.102. 32559359

[jkaf105-B96] Quinlan AR, Hall IM. 2010. BEDTools: a flexible suite of utilities for comparing genomic features. Bioinformatics. 26(6):841–842. doi:10.1093/bioinformatics/btq033. 20110278 PMC2832824

[jkaf105-B97] Ramírez SR, Eltz T, Fujiwara MK, Gerlach G, Goldman-Huertas B, Tsutsui ND, Pierce NE. 2011. Asynchronous diversification in a specialized plant-pollinator mutualism. Science. 333(6050):1742–1746. doi:10.1126/science.1209175. 21940893

[jkaf105-B98] R Core Team . 2024. R: A Language and Environment for Statistical Computing. Vienna, Austria: R Foundation for Statistical Computing. https://www.R-project.org.

[jkaf105-B99] Revell LJ . 2024. Phytools 2.0: an updated R ecosystem for phylogenetic comparative methods (and other things). PeerJ. 12:e16505. doi:10.7717/peerj.16505. 38192598 PMC10773453

[jkaf105-B100] Rice P, Longden I, Bleasby A. 2000. EMBOSS: the European molecular biology open software suite. Trends Genet. 16(6):276–277. doi:10.1016/S0168-9525(00)02024-2. 10827456

[jkaf105-B101] Robertson HM . 2015. The insect chemoreceptor superfamily is ancient in animals. Chem Senses. 40(9):609–614. doi:10.1093/chemse/bjv046. 26354932

[jkaf105-B102] Robertson HM, Wanner KW. 2006. The chemoreceptor superfamily in the honey bee, *Apis mellifera*: expansion of the odorant, but not gustatory, receptor family. Genome Res. 16(11):1395–1403. doi:10.1101/gr.5057506. 17065611 PMC1626641

[jkaf105-B103] Rytz R, Croset V, Benton R. 2013. Ionotropic receptors (IRs): chemosensory ionotropic glutamate receptors in *Drosophila* and beyond. Insect Biochem Mol Biol. 43(9):888–897. doi:10.1016/j.ibmb.2013.02.007. 23459169

[jkaf105-B104] Sánchez-Gracia A, Vieira FG, Rozas J. 2009. Molecular evolution of the major chemosensory gene families in insects. Heredity (Edinb). 103(3):208–216. doi:10.1038/hdy.2009.55. 19436326

[jkaf105-B105] Slater GSC, Birney E. 2005. Automated generation of heuristics for biological sequence comparison. BMC Bioinformatics. 6(1):31. doi:10.1186/1471-2105-6-31. 15713233 PMC553969

[jkaf105-B106] Sobolevsky AI, Rosconi MP, Gouaux E. 2009. X-ray structure, symmetry and mechanism of an AMPA-subtype glutamate receptor. Nature. 462(7274):745–756. doi:10.1038/nature08624. 19946266 PMC2861655

[jkaf105-B107] Stanke M, Keller O, Gunduz I, Hayes A, Waack S, Morgenstern B. 2006. AUGUSTUS: ab initio prediction of alternative transcripts. Nucleic Acids Res. 34(Web Server issue):W435–W439. doi:10.1093/nar/gkl200. 16845043 PMC1538822

[jkaf105-B108] Sun C, Huang J, Wang Y, Zhao X, Su L, Thomas GWC, Zhao M, Zhang X, Jungreis I, Kellis M, et al 2021. Genus-wide characterization of bumblebee genomes provides insights into their evolution and variation in ecological and behavioral traits. Mol Biol Evol. 38(2):486–501. doi:10.1093/molbev/msaa240. 32946576 PMC7826183

[jkaf105-B109] Suyama M, Torrents D, Bork P. 2006. PAL2NAL: robust conversion of protein sequence alignments into the corresponding codon alignments. Nucleic Acids Res. 34(Web Server issue):W609–W612. doi:10.1093/nar/gkl315. 16845082 PMC1538804

[jkaf105-B110] Tabatabaee Y, Zhang C, Warnow T, Mirarab S. 2023. Phylogenomic branch length estimation using quartets. Bioinformatics. 39(39 Suppl 1):i185–i193. doi:10.1093/bioinformatics/btad221. 37387151 PMC10311336

[jkaf105-B111] Vertacnik KL, Linnen CR. 2017. Evolutionary genetics of host shifts in herbivorous insects: insights from the age of genomics. Ann N Y Acad Sci. 1389(1):186–212. doi:10.1111/nyas.13311. 28218960

[jkaf105-B112] Walker BJ, Abeel T, Shea T, Priest M, Abouelliel A, Sakthikumar S, Cuomo CA, Zeng Q, Wortman J, Young SK, et al 2014. Pilon: an integrated tool for comprehensive microbial variant detection and genome assembly improvement. PLoS One. 9(11):e112963. doi:10.1371/journal.pone.0112963. 25409509 PMC4237348

[jkaf105-B113] Wang Y, Fang G, Xu P, Gao B, Liu X, Qi X, Zhang G, Cao S, Li Z, Ren X, et al 2022. Behavioral and genomic divergence between a generalist and a specialist fly. Cell Rep. 41(7):111654. doi:10.1016/j.celrep.2022.111654. 36384127

[jkaf105-B114] Wang Y, Qiu L, Wang B, Guan Z, Dong Z, Zhang J, Cao S, Yang L, Wang B, Gong Z, et al 2024. Structural basis for odorant recognition of the insect odorant receptor OR-Orco heterocomplex. Science. 384(6703):1453–1460. doi:10.1126/science.adn6881. 38870272

[jkaf105-B115] Wappler T, Labandeira CC, Engel MS, Zetter R, Grímsson F. 2015. Specialized and generalized pollen-collection strategies in an ancient bee lineage. Curr Biol. 25(23):3092–3098. doi:10.1016/j.cub.2015.09.021. 26585282 PMC6485464

[jkaf105-B116] Waser NM, Ollerton J. 2006. Plant-pollinator interactions: from specialization to generalization. Chicago, IL: University of Chicago Press.

[jkaf105-B117] Wicher D, Miazzi F. 2021. Functional properties of insect olfactory receptors: ionotropic receptors and odorant receptors. Cell Tissue Res. 383(1):7–19. doi:10.1007/s00441-020-03363-x. 33502604 PMC7873100

[jkaf105-B118] Wood TJ, Müller A, Praz C, Michez D. 2023. Elevated rates of dietary generalization in eusocial lineages of the secondarily herbivorous bees. BMC Ecol Evol. 23(1):67. doi:10.1186/s12862-023-02175-1. 37986035 PMC10662511

[jkaf105-B119] Yang Z . 2007. PAML 4: phylogenetic analysis by maximum likelihood. Mol Biol Evol. 24(8):1586–1591. doi:10.1093/molbev/msm088. 17483113

[jkaf105-B120] Zhang Y, Skolnick J. 2005. TM-align: a protein structure alignment algorithm based on the TM-score. Nucleic Acids Res. 33(7):2302–2309. doi:10.1093/nar/gki524. 15849316 PMC1084323

[jkaf105-B121] Zhao J, Chen AQ, Ryu J, del Mármol J. 2024. Structural basis of odor sensing by insect heteromeric odorant receptors. Science. 384(6703):1460–1467. doi:10.1126/science.adn6384. 38870275 PMC11235583

[jkaf105-B122] Zhao Z, McBride CS. 2020. Evolution of olfactory circuits in insects. J Comp Physiol A Neuroethol Sens Neural Behav Physiol. 206(3):353–367. doi:10.1007/s00359-020-01399-6. 31984441 PMC7192870

[jkaf105-B123] Zhou X, Rokas A, Berger SL, Liebig J, Ray A, Zwiebel LJ. 2015. Chemoreceptor evolution in hymenoptera and its implications for the evolution of eusociality. Genome Biol Evol. 7(8):2407–2416. doi:10.1093/gbe/evv149. 26272716 PMC4558866

