# Supplementary material for: Shifts in bee diet breadths are associated with gene gains and losses and positive selection across olfactory receptors
Source: G3 (Bethesda). 2025 Jul 11;15(8):jkaf105. doi: 10.1093/g3journal/jkaf105 (PMC12341933; doi:10.1093/g3journal/jkaf105)
Supplement: jkaf105_Supplementary_Data [file jkaf105_supplementary_data.zip › Supplementary_Figures_G3-2025-405783.pdf]

Supplementary Figure S1: Maximum likelihood gene tree of ORs annotated across bee species showing assigned orthogroups and indicating bootstrap values. This gene tree was constructed from 4,950 OR gene sequences using ModelFinder in IQTree2 to identify the best-fit substitution model with 1000 bootstrap replicates. Branch support values >= 0.7 are indicated by yellow dots in the middle of branches. Genes were assigned into 47 orthogroups through multiple manual curation steps (See Methods), indicated by labels and yellow shaded panels above clades. The secondary strip indicates diet breadth assignments for species coded by colour: red - broad polylectic species, blue - polylectic species, green - oligolectic species and orange - monolectic species. Given the large number of tips, we omitted tip labels for the gene tree to ensure clarity of interpretation.

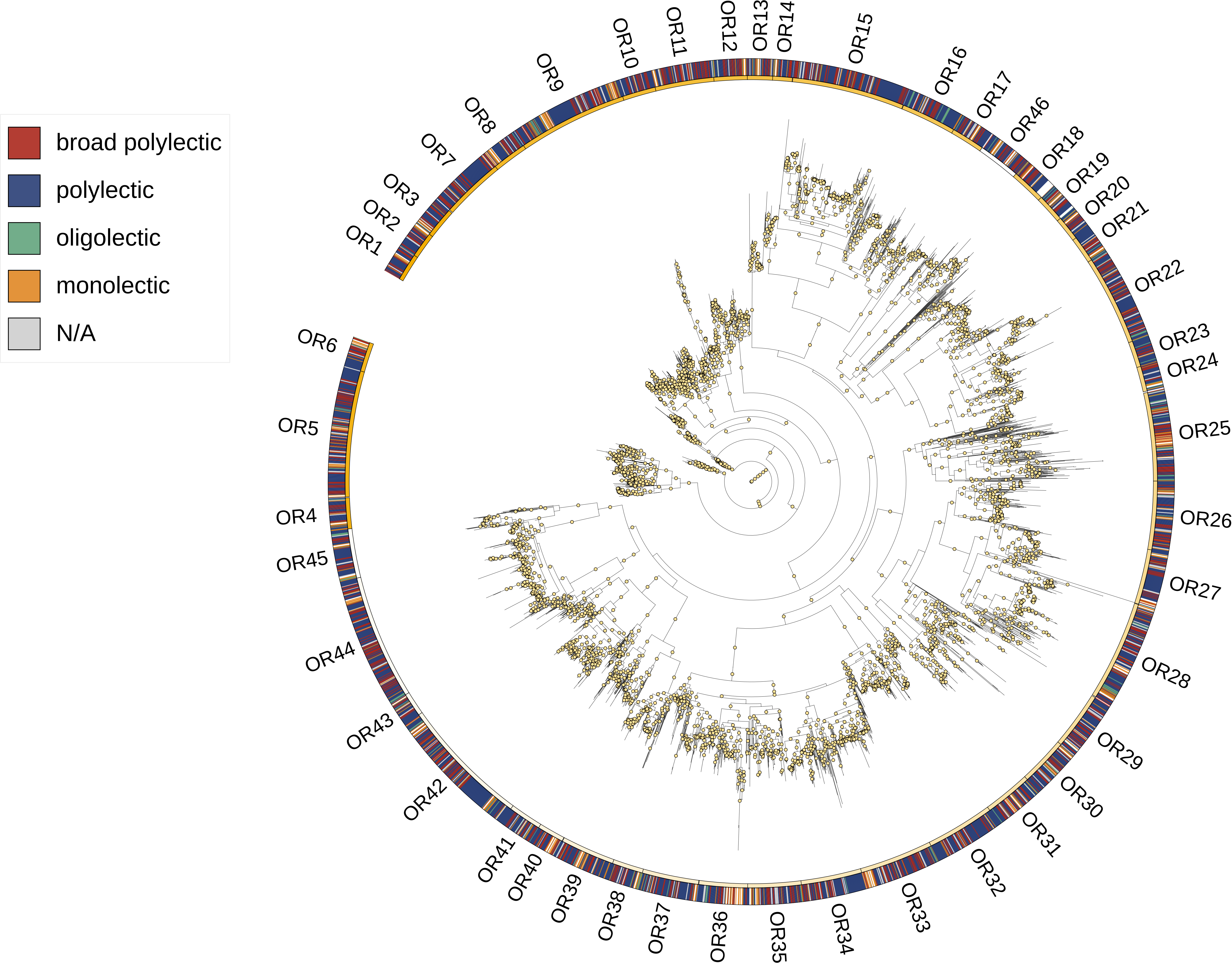
broad polylectic
polylectic
oligolectic
monolectic
N/A
OR1
OR2
OR3
OR7
OR8
OR9
OR10
OR11
OR12
OR13
OR14
OR15
OR16
OR17
OR46
OR18
OR19
OR20
OR21
OR22
OR23
OR24
OR25
OR26
OR27
OR28
OR29
OR30
OR31
OR32
OR33
OR34
OR35
OR36
OR37
OR38
OR39
OR40
OR41
OR42
OR43
OR44
OR45
OR4
OR5
OR6

Supplementary Figure S2: Maximum likelihood gene tree of GRs annotated across bee species showing assigned orthogroups and indicating bootstrap values. This gene tree was constructed from 749 GR gene sequences using ModelFinder in IQTree2 to identify the best-fit substitution model with 1000 bootstrap replicates. Branch support values >= 0.7 are indicated by red dots in the middle of branches. Genes were assigned into 15 orthogroups through multiple manual curation steps (See Methods), indicated by labels and red shaded panels above clades. The secondary strip indicates diet breadth assignments for species coded by colour: red - broad polylectic species, blue - polylectic species, green - oligolectic species and orange - monolectic species.

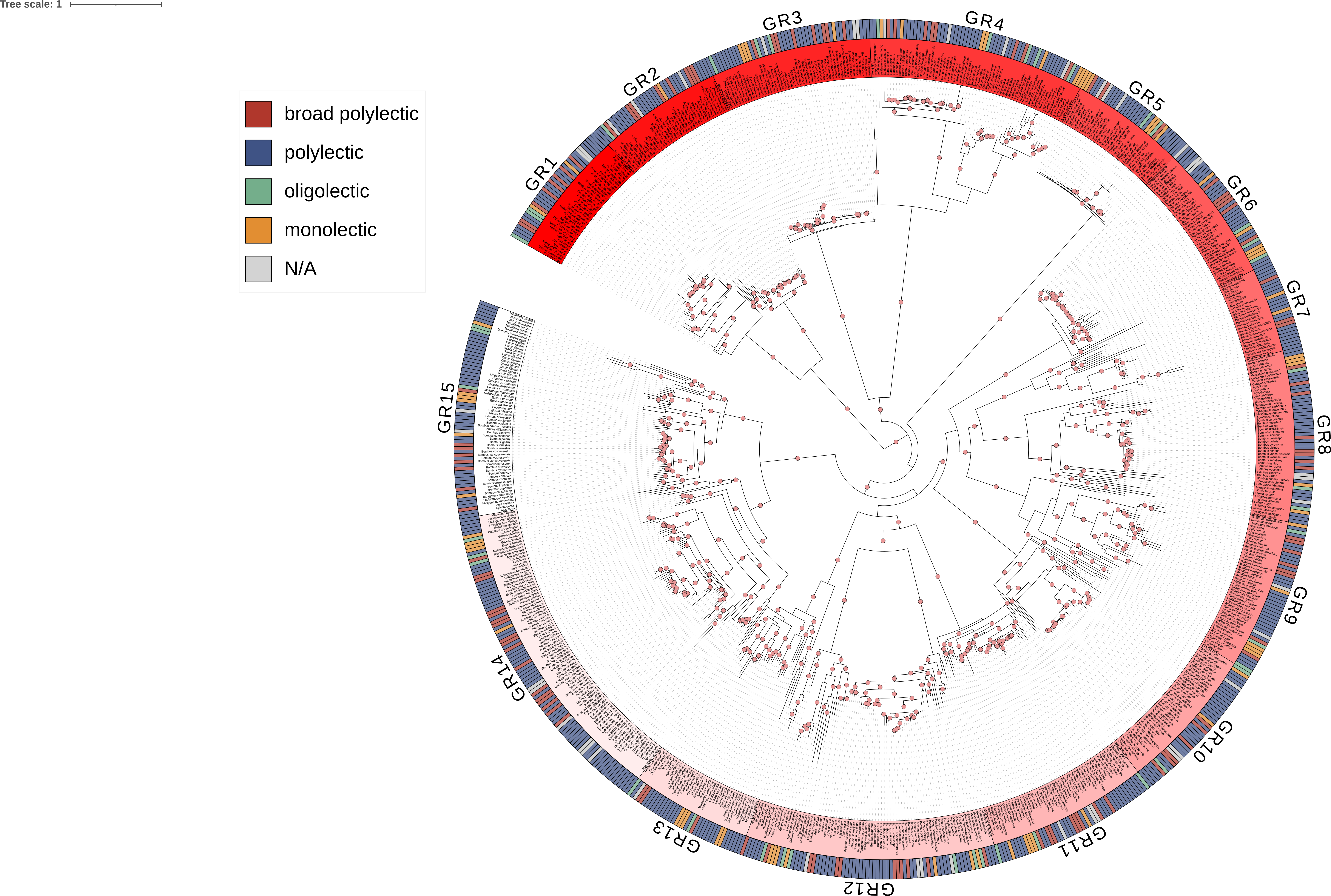
Tree scale: 1
broad polylectic
polylectic
oligolectic
monolectic
N/A
GR1
GR2
GR3
GR4
GR5
GR6
GR7
GR8
GR9
GR10
GR11
GR12
GR13
GR14
GR15

Supplementary Figure S3: Maximum likelihood gene tree of IRs annotated across bee species showing assigned orthogroups and indicating bootstrap values. This gene tree was constructed from 484 IR gene sequences using ModelFinder in IQTree2 to identify the best-fit substitution model with 1000 bootstrap replicates. Branch support values >= 0.7 are indicated by green dots in the middle of branches. Genes were assigned into 9 orthogroups through multiple manual curation steps (See Methods), indicated by labels and green shaded panels above clades. The secondary strip indicates diet breadth assignments for species coded by colour: red - broad polylectic species, blue - polylectic species, green - oligolectic species and orange - monolectic species.

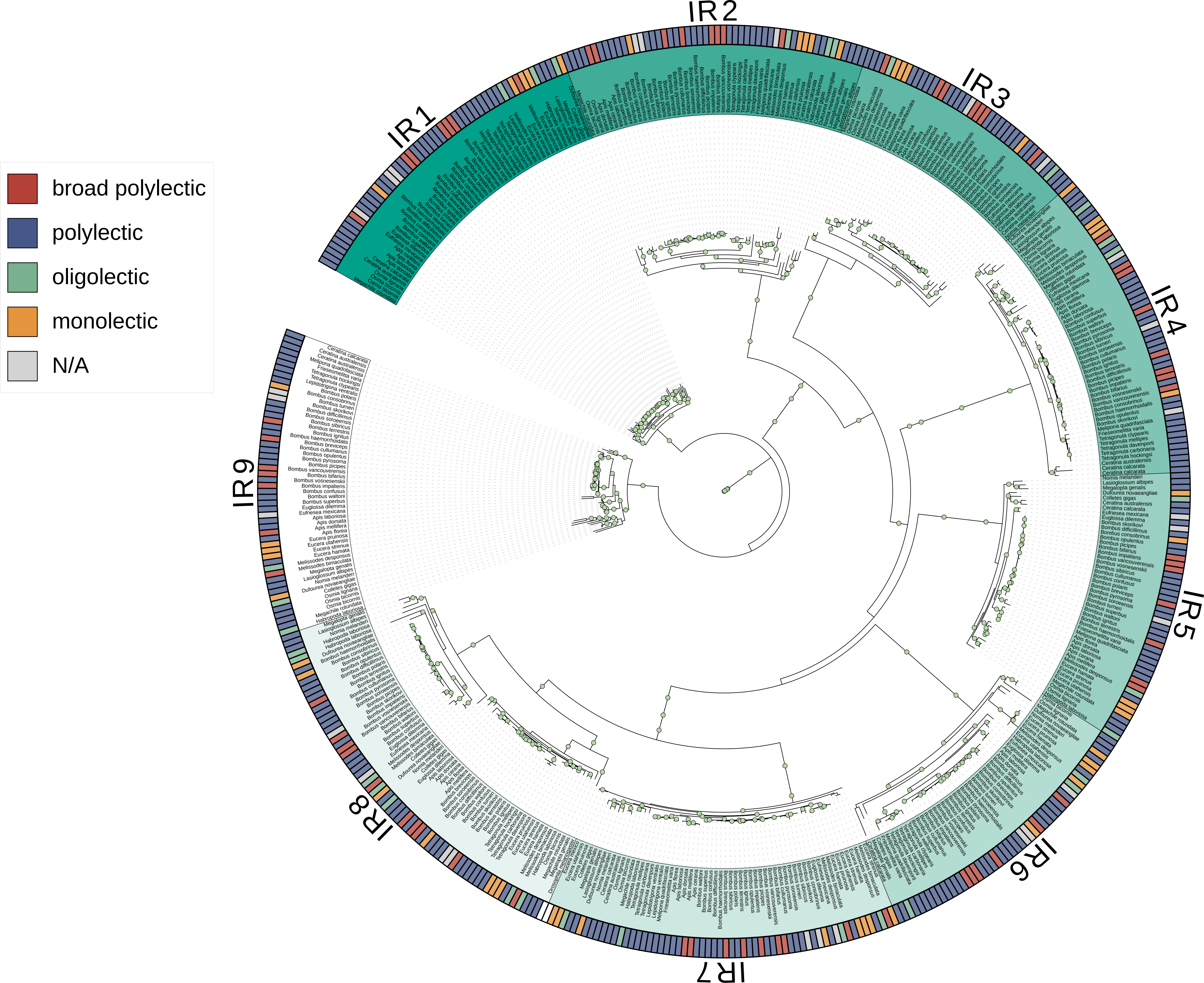
broad polylectic
polylectic
oligolectic
monolectic
N/A
IR1
IR2
IR3
IR4
IR5
IR6
IR7
IR8
IR9

Supplementary Figure S4: 3-D structures of three GR orthogroups showing diversification within specialist bee species, as identified by branch-site models implemented in codeml and HYPHY. 3-D structural models were built in AlphaFold2 and compared to previously generated crystal structures of GR9 proteins to assess completeness. Models were generated for three orthogroups: **(a)** GR1: which showed diversification in monolectic species, **(b)** GR7: which showed diversification in monolectic and oligolectic species and **(c)** GR8: which also showed diversification in monolectic species. Two visualizations of each structural model are shown: **(i)** structures coloured by predicted local distance difference test (pLDDT) values as for Figures 3 and 4 and showing putative extracellular (ECL), transmembrane (TMD) and intracellular (ICL) domains, where applicable, and **(ii)** structures indicating individual protein helices, labelled with transmembrane domain IDs, where applicable. Diversifying codons within monolectic and oligolectic species were identified using FEL-CONTRAST models and represented within each of the structural models as red spheres. Models differed in their completeness – we were able to assign putative helix IDs for the GR1 model and protein domains for the GR1 and GR8 models. The model for GR7 was highly fragmented and we could not conclusively assign domain and helix identities for this structure.

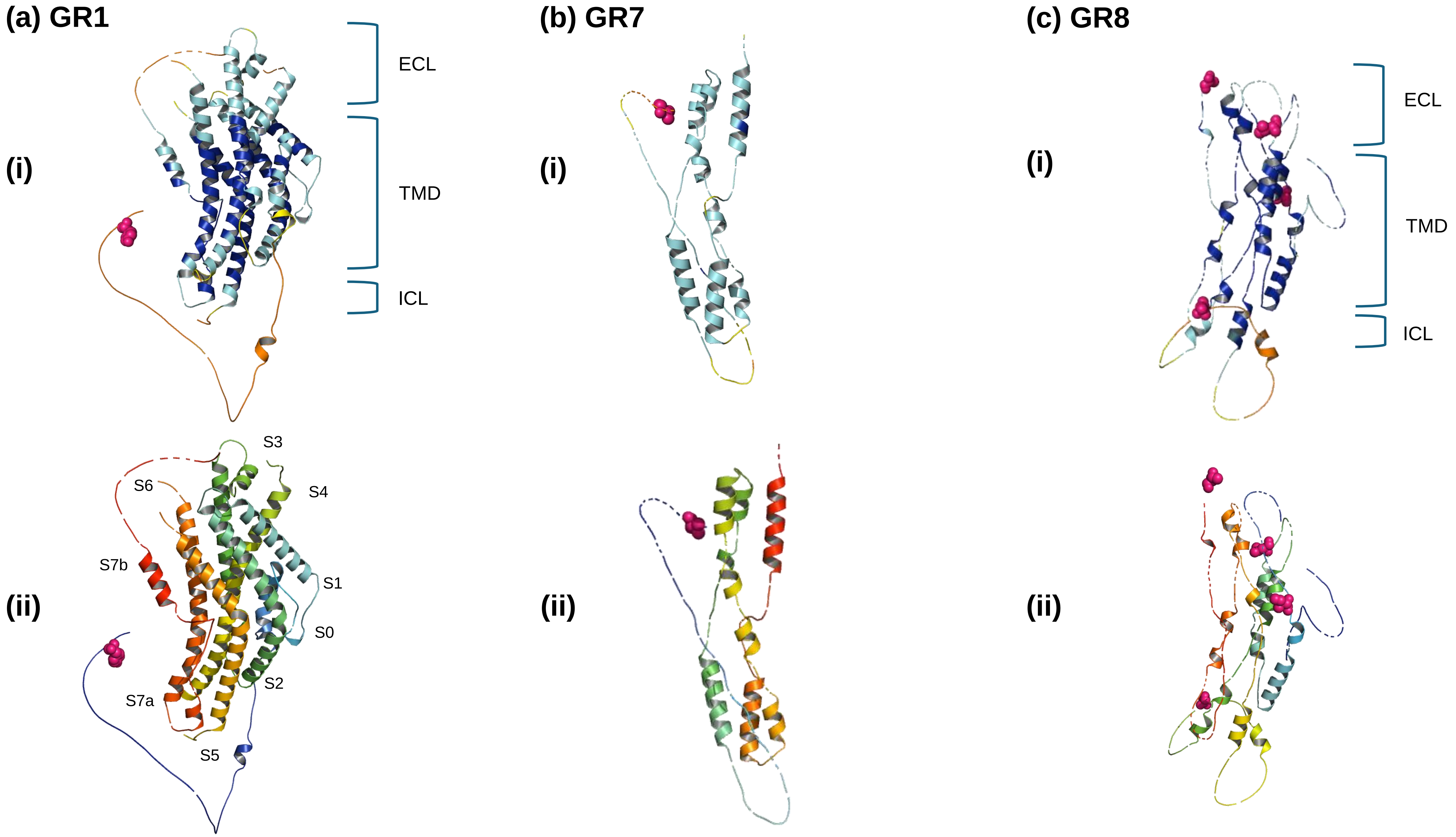

(a) GR1
(b) GR7
(c) GR8
(i)
(ii)
ECL
TMD
ICL
S0
S1
S2
S3
S4
S5
S6
S7a
S7b

Supplementary Figure S5: 3-D structures of three OR orthogroups showing diversification within specialist bee species, as identified by branch-site models implemented in codeml and HYPHY. 3-D structural models were built in AlphaFold2 and compared to previously generated crystal structures of AbOrco proteins to assess completeness. Models were generated for three orthogroups, all of which showed diversification in monolectic and oligolectic species: **(a)** OR3, **(b)** OR3 and **(c)** OR24. Two visualizations of each structural model are shown: **(i)** structures coloured by predicted local distance difference test (pLDDT) values as for Figures 3 and 4 and showing putative extracellular (ECL), transmembrane (TMD) and intracellular (ICL) domains and **(ii)** structures indicating individual protein helices, labelled with transmembrane domain IDs. Diversifying codons within monolectic and oligolectic species were identified using FEL-CONTRAST models and represented within each of the structural models as red spheres. Models differed in their completeness – models for OR3 and OR4 showed seven transmembrane domains, as predicted by previous work but the model for OR24 showed only 6 transmembrane domains.

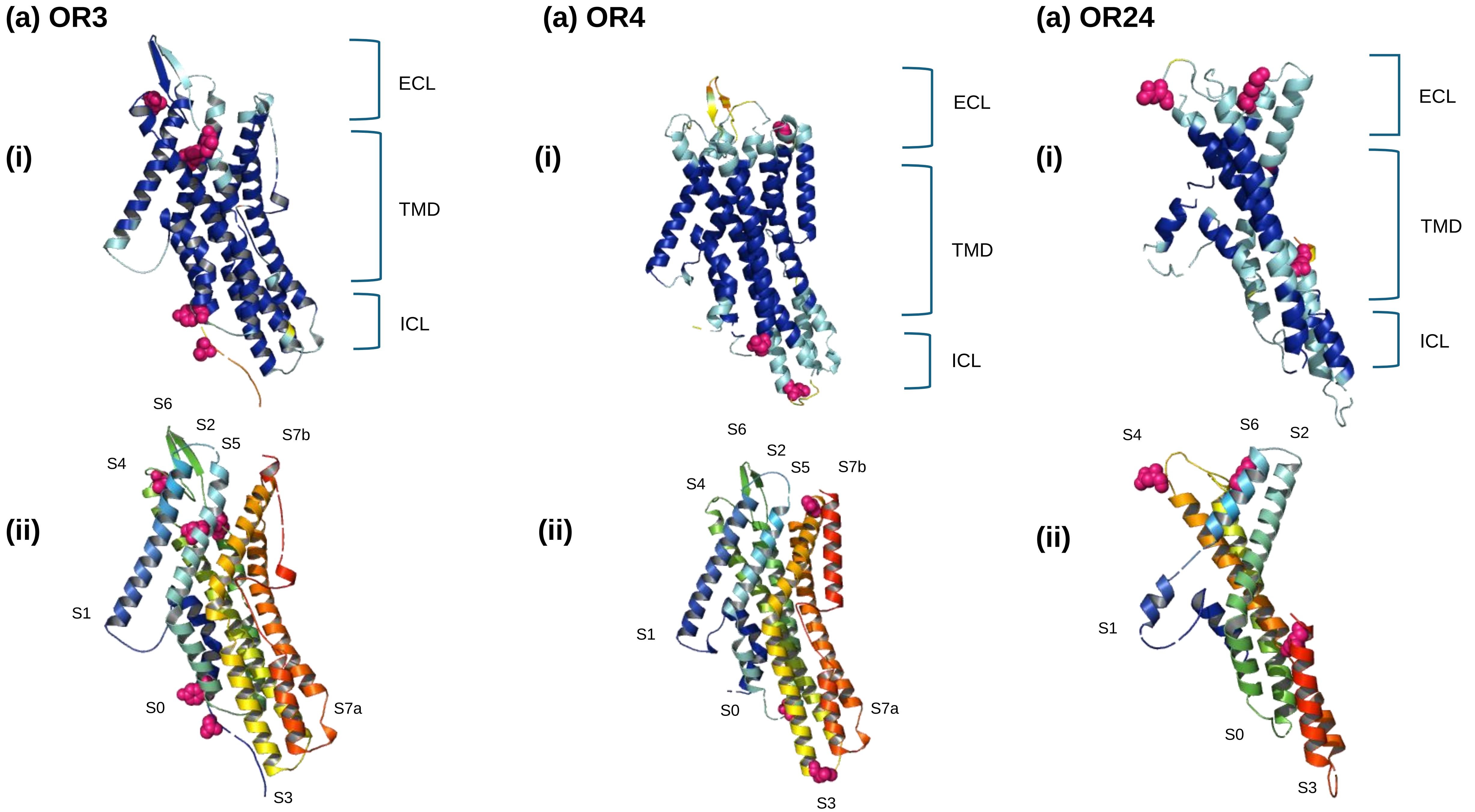

(a) OR3
(a) OR4
(a) OR24
(i)
(ii)
ECL
TMD
ICL
S0
S1
S2
S3
S4
S5
S6
S7a
S7b
