# Supplementary material for: Shifts in bee diet breadths are associated with gene gains and losses and positive selection across olfactory receptors
Source: G3 (Bethesda). 2025 Jul 11;15(8):jkaf105. doi: 10.1093/g3journal/jkaf105 (PMC12341933; doi:10.1093/g3journal/jkaf105)
Supplement: jkaf105_Supplementary_Data [file jkaf105_supplementary_data.zip › Supplementary_Material_Legends_G3-2025-405783.docx]

**Supplemental Information for Singh et al.**

**Supplementary Tables:**

1. Supplementary Table S1: Sample collection information for *de-novo* genomes and antennal transcriptomes
2. Supplementary Table S2: gDNA extractions
3. Supplementary Table S3: Antennal RNA extractions
4. Supplementary Table S4: SRA accession IDs and corresponding citations for previously sequenced bee genomes
5. Supplementary Table S5: BUSCO scores and QUAST genome completeness metrics for assembled genomes
6. Supplementary Table S6: Comparison of BUSCO scores of our assemblies and previously assembled short-read genomes
7. Supplementary Table S7: Diet breadth assignments for species used for this study and corresponding references
8. Supplementary Table S8: Marginal probabilities for each diet breadth category occurring at each node of the phylogeny
9. Supplementary Table S9: Fossils used to calibrate species phylogeny and corresponding references
10. Supplementary Table S10: Comparisons of OR, GR and IR gene repertoires annotated for this study and previous studies
11. Supplementary Table S11: Estimated parameters for gene gain and loss rate calculations and branch-specific gene turnover metrics
12. Supplementary Table S12: Phylogenetic signal in continuous variables
13. Supplementary Table S13: Estimated coefficients of PGLS models
14. Supplementary Table S14: Pruned dataset used to re-run gain/loss analyses and estimated model coefficients
15. Supplementary Table S15: Estimated rates of molecular evolution (dN/dS, PAML branch-site)
16. Supplementary Table S16: Estimated rates of molecular evolution (dN/dS, HYPHY BUSTED-Ph)
17. Supplementary Table S17: tBLASTn results for each orthogroup identified as experiencing positive selection
18. Supplementary Table S18: Metrics assessing completeness of AlphaFold2 structural models
19. Supplementary Table S19: Site-based variation in molecular evolution rates (dN/dS, HYPHY CONTRAST-FEL)

**Supplementary Figures:**

1. Supplementary Figure S1: Phylogeny of ORs showing 46 orthogroups
2. Supplementary Figure S2: Phylogeny of GRs showing 15 orthogroups
3. Supplementary Figure S3: Phylogeny of IRs showing 9 orthogroups
4. Supplementary Figure S4: AlphaFold2 structures for three GR orthogroups experiencing positive selection
5. Supplementary Figure S5: AlphaFold2 structures for three OR orthogroups experiencing positive selection
